# Liquid biopsy at the frontier in renal cell carcinoma: recent analysis of techniques and clinical application

**DOI:** 10.1186/s12943-023-01745-7

**Published:** 2023-02-21

**Authors:** Mingyang Li, Lei Li, Jianyi Zheng, Zeyu Li, Shijie Li, Kefeng Wang, Xiaonan Chen

**Affiliations:** Department of Urology, Shengjing Hospital of China Medical University, No. 36 Sanhao Street, Heping District, Liaoning Shenyang, 110004 People’s Republic of China

**Keywords:** Renal cell carcinoma, Liquid biopsy, Circulating tumor cells, Cell-free tumor DNA, Diagnosis, Prognosis, Treatment monitoring

## Abstract

Renal cell carcinoma (RCC) is a major pathological type of kidney cancer and is one of the most common malignancies worldwide. The unremarkable symptoms of early stages, proneness to postoperative metastasis or recurrence, and low sensitivity to radiotherapy and chemotherapy pose a challenge for the diagnosis and treatment of RCC. Liquid biopsy is an emerging test that measures patient biomarkers, including circulating tumor cells, cell-free DNA/cell-free tumor DNA, cell-free RNA, exosomes, and tumor-derived metabolites and proteins. Owing to its non-invasiveness, liquid biopsy enables continuous and real-time collection of patient information for diagnosis, prognostic assessment, treatment monitoring, and response evaluation. Therefore, the selection of appropriate biomarkers for liquid biopsy is crucial for identifying high-risk patients, developing personalized therapeutic plans, and practicing precision medicine. In recent years, owing to the rapid development and iteration of extraction and analysis technologies, liquid biopsy has emerged as a low cost, high efficiency, and high accuracy clinical detection method. Here, we comprehensively review liquid biopsy components and their clinical applications over the past 5 years. Additionally, we discuss its limitations and predict its future prospects.

## Background

Kidney cancer is a common, substantial lesion of the kidney, accounting for approximately 2.2% of all cancer incidences, and its occurrence continues to increase [1]. Renal cell carcinoma (RCC) is the main pathological type of kidney cancer and constitutes over 90% of all kidney cancers. RCC can be classified into three distinct types based on specific histopathological and genetic features, of which clear-cell RCC (ccRCC) is the most common (80–90%), followed by papillary and suspicious cell carcinoma (15% and 5%, respectively) [2]. Surgery is the preferred treatment for RCC; however, 20–40% of patients with RCC experience recurrence and metastasis after surgery [3]. Therapeutic strategies based on radiation and cytotoxic chemotherapy agents have limited therapeutic effects on RCC [3, 4]. The advent and development of targeted agents, including agents targeting mammalian target of rapamycin (mTOR) and receptor tyrosine kinase signaling, have made therapeutic advances, but are prone to drug resistance [5–8]. In recent years, the prognosis of patients with RCC has improved significantly with the introduction of immune checkpoint inhibitor (ICI) therapy [9]. Many factors induce metastasis and recurrence of RCC and lead to death, with the impact of late diagnosis and lack of effective disease surveillance and drug efficacy prediction methods being the most notable. Therefore, the development of a non-invasive screening tool and the identification of appropriate biomarkers for RCC are urgently necessary.

**Fig. 1:**
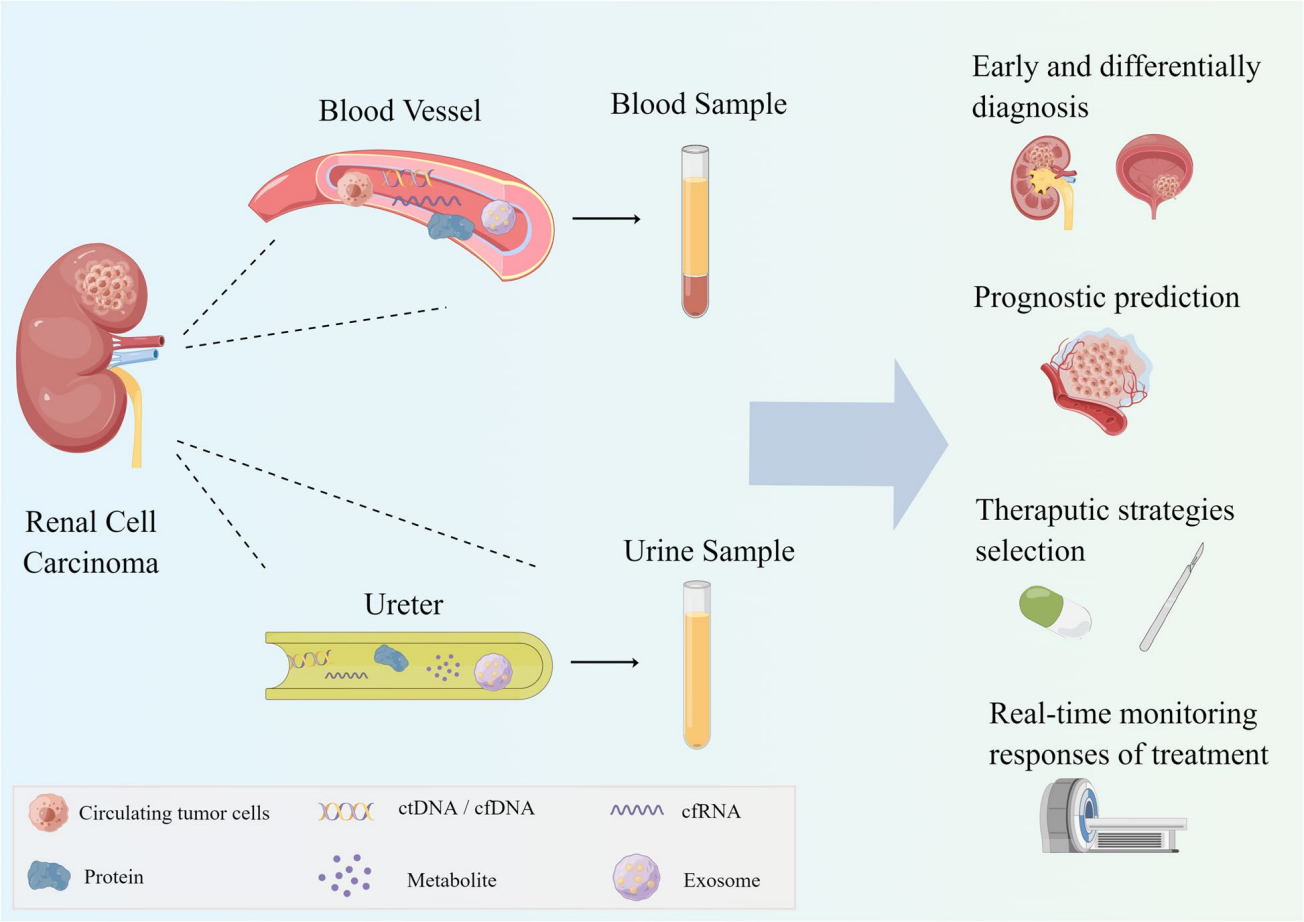
Samples and components for liquid biopsy of RCC. Liquid biopsy for RCC mainly uses blood and urine samples comprising CTCs, cfDNA/ctDNA, cfRNA, proteins, metabolites, and exosomes. Due to its non-invasiveness, liquid biopsy can be used to collect patient information continuously and plays multiple roles at different stages of disease. It can screen patients with RCC in the healthy population and identify urological masses for differential diagnosis. Before treatment, liquid biopsy can predict the risk of progression to identify high-risk patients and predict the response of patients to various treatments, which helps to select the appropriate treatment plan. After treatment, liquid biopsy allows real-time monitoring of patient outcomes and prevention of postoperative recurrence and metastasis

The concept of “liquid biopsy”, a non-invasive exanimation method, has attracted increasing attention [10, 11]. Through collecting and analyzing circulating tumor cells (CTCs) and other tumor markers—cell-free tumor DNA (ctDNA) or circulating DNA (cfDNA), cell-free RNA (cfRNA), exosomes, and tumor derived metabolites and proteins in the blood, urine, pleural fluid, and ascites—liquid biopsy has been widely used in cancers such as liver cancer [12], lung cancer [13], ovarian cancer [14], bladder cancer [15], and other cancers. In RCC, blood and urine are the main samples for liquid biopsy (Fig. 1). With the advantage of non-invasiveness, liquid biopsies can be used to continuously collect samples from patients to detect disease progression or predict drug efficacy, which has the potential to be developed as a screening tool compared to conventional tissue biopsies, which can only provide localized lesions with more invasiveness.

In recent years, the rapid update and iteration of technical means in liquid biopsy have made it available for wide application in the clinical diagnosis of RCC. Therefore, liquid biopsy may become a routine method for the diagnosis and prediction of RCC prognosis in the future. Here, we review the technical methods and clinical applications of liquid biopsy in RCC over the past 5 years.

### Components for liquid biopsy

**Fig. 2:**
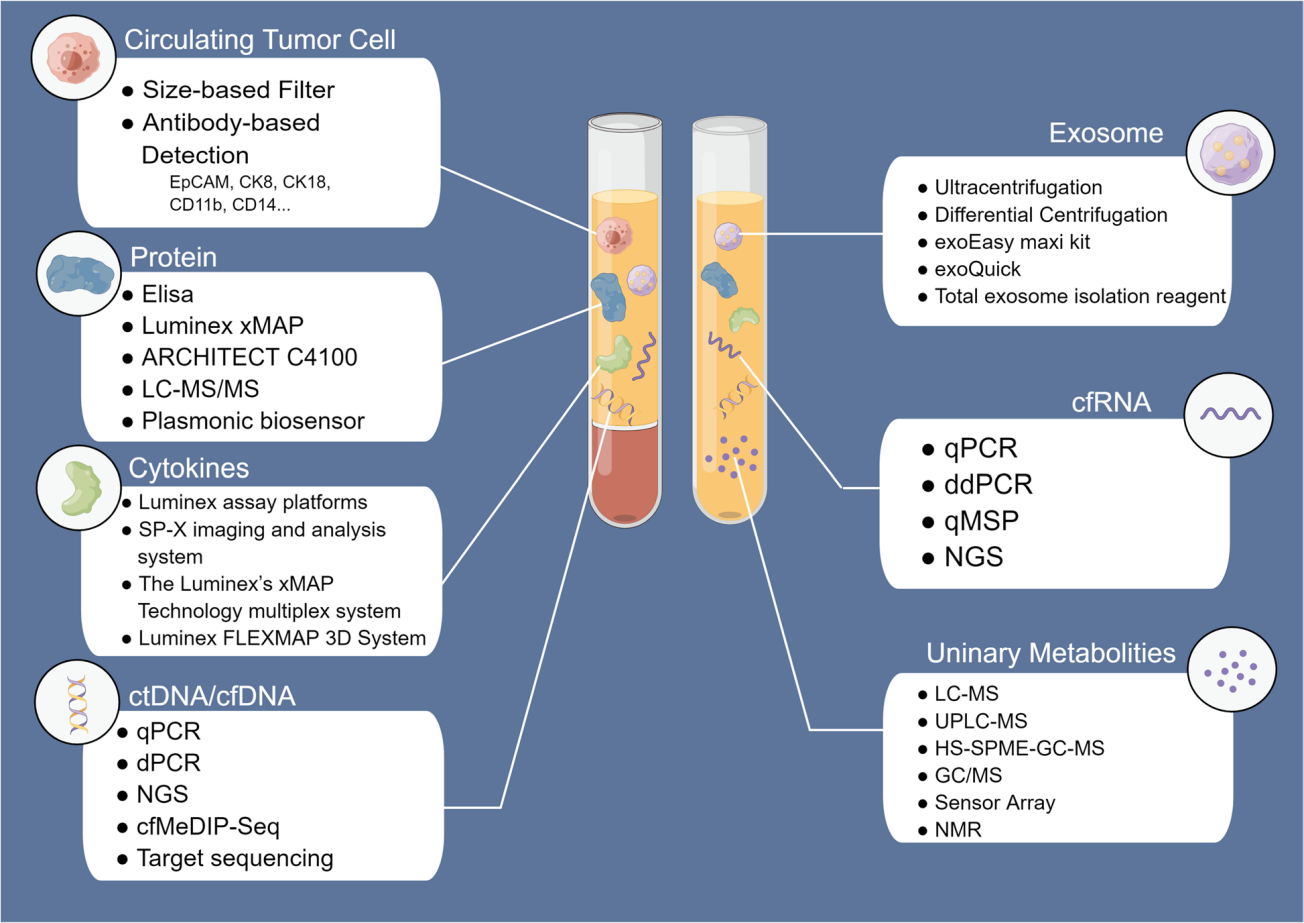
Commonly used techniques for extraction or analysis of RCC liquid biopsies in recent years. CTC isolation mainly includes size-based and antibody-based methods. cfDNA and ctDNA are separated by patients’ genomic alterations. PCR and sequencing are commonly used to detect and analyze mutations, size, expression, and methylation levels. ddPCR and targeting sequencing enable analysis of specific rare DNA with high sensitivity. With the development and diffusion of NGS, researchers can perform high-throughput analysis of cfDNA/ctDNA at a reduced cost. Similar to cfDNA/ctDNA, cfRNA is commonly analyzed by qPCR, ddPCR, methylation-specific quantitative PCR (qMSP), and NGS. Metabolite analysis is mainly performed using MS-based methods, while NMR and inductors have also been used in recent years. Protein analysis mainly depended on ELISA, the standard method for protein level measurements. Some automated analyzers with low cost and high efficiency are commercially available, which have potential for large-scale clinical applications. As an important field of proteomics, circulating cytokine assays use commercial detection platforms or technologies more frequently than Elisa, which enable a rapid detection of multiple cytokines in the blood. Up to now, there is no standard method for the extraction of exosomes. The most commonly used methods in recent years are ultracentrifugation and differential centrifugation. Meanwhile, some extraction reagents are used, such as exoQuick kit, exoEasy maxi kit and Total exosome isolation reagent

Collection of blood or urine from patients with RCC and subsequent analysis of the status of CTCs, ctDNA, cfRNA, proteins, metabolites, and exosomes can enable the construction of clinical models for the screening, disease monitoring, differential diagnosis, and treatment evaluation of Patients with RCC. This in turn can contribute to the selection of treatment strategies and the practice of personalized medicine. Commonly used probes include those for gene expression and mutation, protein levels, and nucleic acid methylation status (Fig. 2).

### CTCs

CTCs are cancer cells that circulate in the peripheral blood after being naturally excreted from primary or metastatic tumors. Despite their small quantities, CTCs can promote tumor metastasis and progression, which may play a major role in tumor metastasis and recurrence [16, 17]. CTCs have shown considerable clinical value in various cancers [18, 19].

**Fig. 3:**
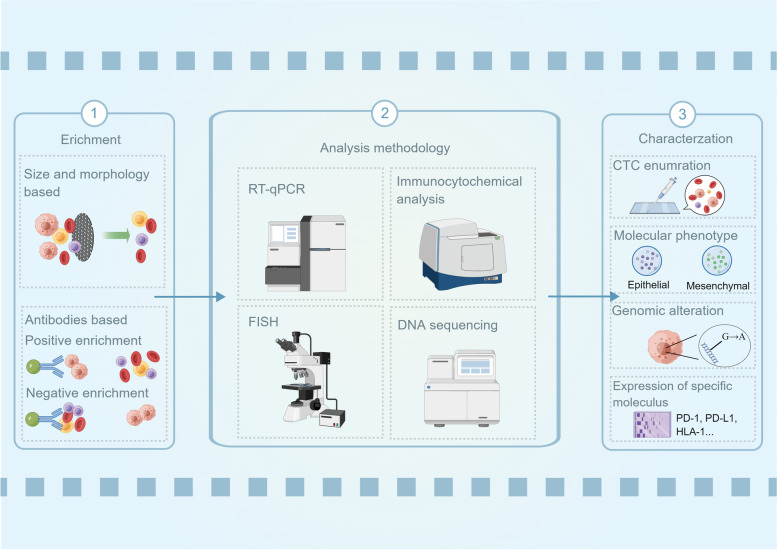
Isolation, detection, and characterization of CTCs and the associated clinical value. CTCs are firstly isolated from peripheral blood by size-, morphology-, and antibody-based methods. Then, several methods can be used to detect CTCs from isolating products or further characterize CTCs by analyzing cellular components or morphology. CTC analysis can provide a range of information such as CTC enumeration, molecular phenotypes, mutations, proteomics and metabolomics to analyze patient heterogeneity, drug resistance and risk of progression, which is widely used for early and differential diagnosis, prognostic assessment, treatment schedule selecting, and evaluation of treatment efficacy

CTC analysis can be divided into enrichment, detection, and characterization (Fig. 3) [20]. No standardized enrichment method for CTCs currently exists. Common CTC enrichment methods can be classified as antibody-based, size-based, and density-based. In the last 5 years, epithelial cell adhesion molecule (EpCAM)-based antibody enrichment methods have become very common in CTC analysis using magnetic beads with antibody functions [21, 22]. The CellSearch system, developed based on EpCAM antibodies, is the first and only FDA-approved clinical method for capturing and counting CTCs [23]. In RCC, the use of a single EpCAM antibody has limited enrichment capacity for CTCs; therefore, carbonic anhydrase IX, XII (CAIX, CAXII), and cytokeratin antibodies were used to capture CTCs in conjunction with EpCAM antibodies [24, 25]. Moreover, multiple clusters of differentiation (CD)-based antibodies were used to identify and exclude other cells, such as myeloid cells (CD11b and CD14) and plasma B cells (CD235a) [24]. In addition, size-based isolation methods have frequently been used in recent years. Filtration membrane-based separations mainly capture cells larger than 8 μm, which can reduce the loss of CTCs and improve their utilization rate [26, 27]. Currently, in addition to the CellSearch System, a variety of technologies, platforms, and products have been developed according to the two methods, including the VERSA Platform [28], FISHMAN-R flow cytometer (On-Chip Biotechnologies, Japan), and Can Patrol CTC enrichment technique (SurExam, China), which were developed according to the antibody-based method, and the CTC-BIOPSY system (Youzhiyou, China) and ISET [29], which were developed according to the size-based method. A previous study compared the capture efficiency of the CellSearch System and ISET for CTCs and concluded that ISET was more suitable for CTC isolation in RCC [30]. CTC detection is mainly achieved using immunocytochemical analysis, including immunohistochemical staining and immunofluorescence. Morphological or image analysis is mainly used as supplementary analysis for immunohistochemical staining. In addition to enumeration, CTCs can also be further characterized by measuring the expression and mutation of specific genes or proteins for more accurate evaluation. Common methods include immunohistochemical analysis, quantitative polymerase chain reaction (qPCR), fluorescence in situ hybridization, and sequencing.

Notably, several new enrichment methods for CTCs in RCC have been proposed to improve CTC isolation efficiency, such as slit-filter-based CTC isolation methods and antibody-based enrichment methods using CD45-negative/G250-positive expression; however, high-quality studies with large sample sizes are needed to test their efficacy [31–34].

### cfDNA/ctDNA

Normally, DNA in plasma is referred to as cfDNA, which is released by necrotic or apoptotic cells, and the DNA released by CTCs is called ctDNA [35–37]. Compared with cfDNA, ctDNA levels are very low and contain smaller fragments [38, 39]; as such, it has been reported that the analysis of cfDNA is more suitable for liquid biopsies [40]. In RCC, a study reported that the fragment size of ctDNA correlates with patient prognosis and has a significant clinical value [41]. ctDNA can only be distinguished from cfDNA based on tumor-specific genomic alterations, which hinders ctDNA/cfDNA analysis. Moreover, the short half-life of cfDNA, which can only exist from 16 min to 2.5 h, makes its extraction quite difficult [42].

DNA molecules contain considerable biological information, including variant sites, expression levels, fragment sizes, and methylation levels. Many studies have focused on VHL, a tumor suppressor that suppresses the progression of RCC through the hypoxia-inducible factor (HIF)/VHL pathway, which is thought to be closely associated with hereditary RCC [43, 44]. The cfDNA research methods include real-time qPCR (RT-qPCR), digital PCR (dPCR), and DNA sequencing. As a relatively traditional method, RT-qPCR can determine information such as the expression, mutation, and methylation levels of target DNA molecules. Compared to RT-qPCR, dPCR, including droplet digital PCR (ddPCR) and BEAMing, has higher sensitivity and applicability for rare ctDNA [45]. DNA sequencing includes targeted sequencing and next-generation sequencing (NGS) for whole-gene sequencing. The former possesses higher sensitivity, while the latter has a broader detection range [46].

Mutation analysis is currently the most widely used method for ctDNA detection through the detection of single nucleotide variation (SNV), copy number variation, indel (insertion-deletion), and fusions. Several studies have shown that some cfDNA/ctDNA has high mutation frequency in patients with RCC, such as VHL, TP53, BAP1, and PBRM1 [47–51]. However, a study detecting the SNV and indels of ctDNA showed that the low mutational frequency of a single ctDNA may not be sufficient for liquid biopsy [48]. Therefore, comprehensive utilization of multiple ctDNA variation statuses, or a combination with other information such as cfDNA/ctDNA levels or fragment size, may be more feasible for clinical use. Moreover, it has become common to calculate microsatellite instability and tumor mutation burden (TMB) based on DNA variation to predict patient prognosis and drug responses independently or in combination with other mutational analyses [52, 53]. Some studies have focused on the calculation of TMB of ctDNA/cfDNA, called blood TMB (b-TMB), rather than the tissue TMB (t-TMB) used in traditional biopsies. Most of these studies have been conducted on lung cancer and other cancers, but not on RCC [54–57]. Although many studies have reported significant correlations between b-TMB and patient clinical progression and drug response, some studies have shown b-TMB is inconsistent with t-TMB [58, 59]. In recent years, with the development of epigenetics, the pathogenic mechanisms of DNA methylation have been explored further. Using cell-free methylated DNA immunoprecipitation and high-throughput sequencing (cfMeDIP-seq) assays and target sequencing, researchers can measure the methylation levels of specific or untargeted DNA, which have gradually shown their high sensitivity and specificity and have the potential to become key clinical indicators [60, 61]. Regarding the sample selection, it has been demonstrated that ctDNA levels are higher in plasma than in serum and are, therefore, more suitable for DNA analysis [62]. Notably, cfDNA is also present in urine, possibly produced by the direct entry of tumor cells or their breakdown products into the urinary tract [63].

### cfRNA

In contrast to cfDNA or ctDNA, cfRNA does not enter the circulation after cell lysis but is secreted by normal and tumor cells [64–66]. Owing to the shortened cell cycle and elevated metabolic level of tumor cells, they produce and secrete more transcripts into the blood or urine [11]. The stability of RNA is lower than that of DNA because of many biological factors, such as RNA hydrolases and metal ions [67–69]. Compared to naked RNA, cfRNA binds to proteins and lipoproteins, which can improve its stability [70, 71], with a half-life of minutes to hours [72], whereas naked RNA is only stable for about 15 s [73]. Meanwhile, some RNAs are encapsulated by extracellular vesicles (EVs) secreted into the circulation by cells, such as exosomes, whose half-life is also significantly higher than that of naked RNA [74, 75].

The vast majority of studies in the last 5 years have focused on the clinical application of microRNAs (miRNAs) rather than mRNAs in liquid biopsies. miRNAs have been shown to play a regulatory role in RCC by promoting or inhibiting tumorigenesis and progression [76–78]. Previously, no single miRNA was found to be sufficiently sensitive and specific to be used as a biomarker for RCC. Therefore, panels consisting of multiple miRNAs are often used as models for RCC diagnosis and evaluation. The methods used to analyze cfRNA were similar to those used to analyze cfDNA. Currently, qPCR and ddPCR are mostly used to verify the expression levels. Notably, in the last 5 years, methylation level analysis was also used with cfRNA, but the result was not as significant as that of DNA [79]. This may be because the experiment was performed only for a single miRNA.

In addition to miRNAs, various other non-coding RNAs have been identified as regulators of RCC and have been explored for their clinical value as biomarkers for liquid biopsies. Long non-coding RNAs (lncRNAs) are a class of non-coding RNAs consisting of more than 200 nucleotides that modulate gene transcription by recruiting chromatin-modifying complexes [80]. Moreover, they interact with mRNAs, miRNAs, and proteins to construct a complex network that also regulates gene transcription and post-transcriptional modifications [81]. Many studies have demonstrated that lncRNAs can influence the tumorigenesis and progression of RCC, such as PVT1 and RCAT1 [82, 83]. Some studies have suggested that lncRNAs have a stronger specificity than other RNA species; therefore, they are more suitable biomarkers in liquid biopsies [84]. Notably, the FDA has approved the detection of PCA3 in urine as a diagnostic method for early prostate cancer, which further emphasizes the significant potential of blood or urine lncRNAs [85]. Circular RNAs (circRNAs) are another class non-coding RNA derived from back splicing and have a wide range of functions, such as targeting and regulating downstream miRNAs as well as influencing the transcription and splicing of parental genes [86]. As circRNAs have become important targets for exploring cancer mechanisms in recent years, an increasing number of differentially expressed circRNAs have been identified in RCC and identified to mediate cancer growth, metastasis, and invasion [87, 88]. It has been suggested that they are highly abundant in multiple body fluids and have a long half-life owing to their resistance to RNase. PIWI-interacting RNAs (piRNAs) are a class of small non-coding RNAs comprising 24–31 nucleotides that have recently been identified in germ cells [89]. Several studies have demonstrated its regulatory role in various cancers. One study showed that overexpression of piR-32051, piR-39894, and piR-43607 was associated with metastasis and worse overall survival in RCC, but their specific functions need to be further clarified [90]. Recently, several experiments have attempted to detect the differential expression of circulating piRNAs between RCC and normal populations, but the possibility of using liquid biopsy markers needs to be explored in more studies [91, 92].

### Metabolomics and proteomics

Cellular metabolites have long been thought to participate in and modulate biosynthetic pathways [93, 94] and regulate biological behavior as signaling molecules [95, 96]. Previous studies have shown that multiple enzymes related to metabolic pathways are involved in the pathogenesis of kidney cancer, such as ferredoxin hydratase (FH) and succinate dehydrogenase (SDH) [97–99]; changing the response of tumors to environmental alterations. Therefore, the detection of changes in circulating metabolite profiles can reveal cancer-induced abnormalities in biometabolic pathways and associated biological behaviors, thus effectively differentiating Patients with RCC from healthy populations, distinguishing ccRCC from other subtypes of RCC, and evaluating treatment efficiency and prognosis. Currently, the analysis of metabolites mainly depends on mass spectrometer (MS)-based methods, including liquid chromatography-MS (LC–MS), gas chromatography-MS (GC–MS), matrix-assisted laser desorption/ionization-MS (MALDI), and related derivative techniques such as ultra-performance LC–MS (UPLC-MS) or headspace solid-phase microextraction coupled with GC–MS (HS–SPME–GC–MS) [100–104]. By accurately measuring compound masses and inferring chemical composition and structure, MS has shown high sensitivity and specificity for probing compounds, becoming the preferred method for structural identification of analytes in complex mixtures. Moreover, nuclear magnetic resonance (NMR) is a commonly used method for the analysis of metabolites [105]. Compared to MS, NMR has lower sensitivity and specificity but can provide more detailed structural information. However, metabolite profiles vary widely among individuals, and although some compounds, such as 2-hydroxyglutarate (2HG), have been co-detected in several clinical trials [100, 106], the panel of compounds used in most experiments is inconsistent.

Human blood and urine are rich in proteins, including carcinoembryonic antigens (CEA), tissue-specific secreted proteins, and intracellular proteins released by tissue injury or death [107, 108]. Similar to metabolite profiling, cancer-associated proteins frequently play an important role in the activation of specific tumorigenesis pathways; thus, their detection can reveal the mechanisms of carcinogenesis or be applied as therapeutic targets. Examples include 78 kDa glucose-regulated protein (GRP78), which affects cellular stress, and programmed cell death protein 1 (PD-1) and programmed cell death ligand 1 (PD-L1), which regulate immune checkpoints (ICs) [109–111]. Therefore, proteomics has the potential to identify important biomarkers in liquid biopsies. Several proteins, such as methemoglobin and CEA, have been used for cancer screening [112, 113], and one of the most widely studied proteins in RCC is kidney injury molecule 1 (KIM 1) [114–116]. Currently, multiple cytokines are tested to assess and monitor the efficacy of treatment in patients with RCC. In addition, as direct regulators of immune function, several chemokines have been used to evaluate the response to immunotherapy and anti-angiogenic therapy, especially IL-6 and IL-8 [117, 118]. In RCC, IL-6 specifically targets IL-6R and gp130 on the cell surface [119], thereby activating various cancer-related signaling pathways, such as the JAK-STAT pathway [120]. Meanwhile, IL-8 binds to IL-8R to recruit immune cells to influence the tumor microenvironment (TME), thereby inhibiting anti-tumor immune function [121]. In addition, as a direct therapeutic target, serum VEGF levels can directly reflect the therapeutic responses and drug resistance of patients, which has been adopted in many studies [122]. In addition, other cytokines such as HGF, IFN-α, and TNF-α have also been widely tested for their clinical value. In the last 5 years, most clinical trials have used enzyme-linked immunosorbent assay (ELISA) to determine protein levels, which is currently the gold standard tool [123]. ELISA can quantify proteins with relatively high sensitivity and a wide dynamic detection range [124]. However, ELISA requires manual operation using kits, and its detection efficiency cannot meet the needs of wide clinical applications, which entails the development and promotion of automatic analysis technology and platforms. The Luminex xMAP system is an automated protein detection platform combining the sandwich immunoassay format with flow cytometry, with a general sensitivity in the range of 1–10 pg/mL and a detection range of approximately 3–4 orders of magnitude [125, 126]. Similarly, the ARCHITECT C4100 Analyzer (Abbott France SA, France) is an automatic immunoassay system that is highly concordant with ELISA for the detection of neutrophil gelatinase-associated lipocalin (NGAL) in urine [127]. In addition, Hu et al. developed a plasma biosensor based on gold nanorods with high refractive index sensitivity. This method has a detection range of 50 pg/mL to 5 mg/mL and is less influenced by admissibility factors, making it promising for application in routine clinical examination [128]. In cytokine detection, researchers are mainly adopting commercially available platforms or products instead of ELISA to improve both the efficiency and convenience to meet the needs of clinical practice. Common analysis platforms or techniques for cytokine includes Luminex assay platforms (Assay Gate, USA), Luminex immune-bead technology and a high-sensitivity kit (Invitrogen/Biosource, USA), Luminex FLEXMAP 3D System (Fisher-Scientific, USA), etc.

### Exosomes

**Fig. 4:**
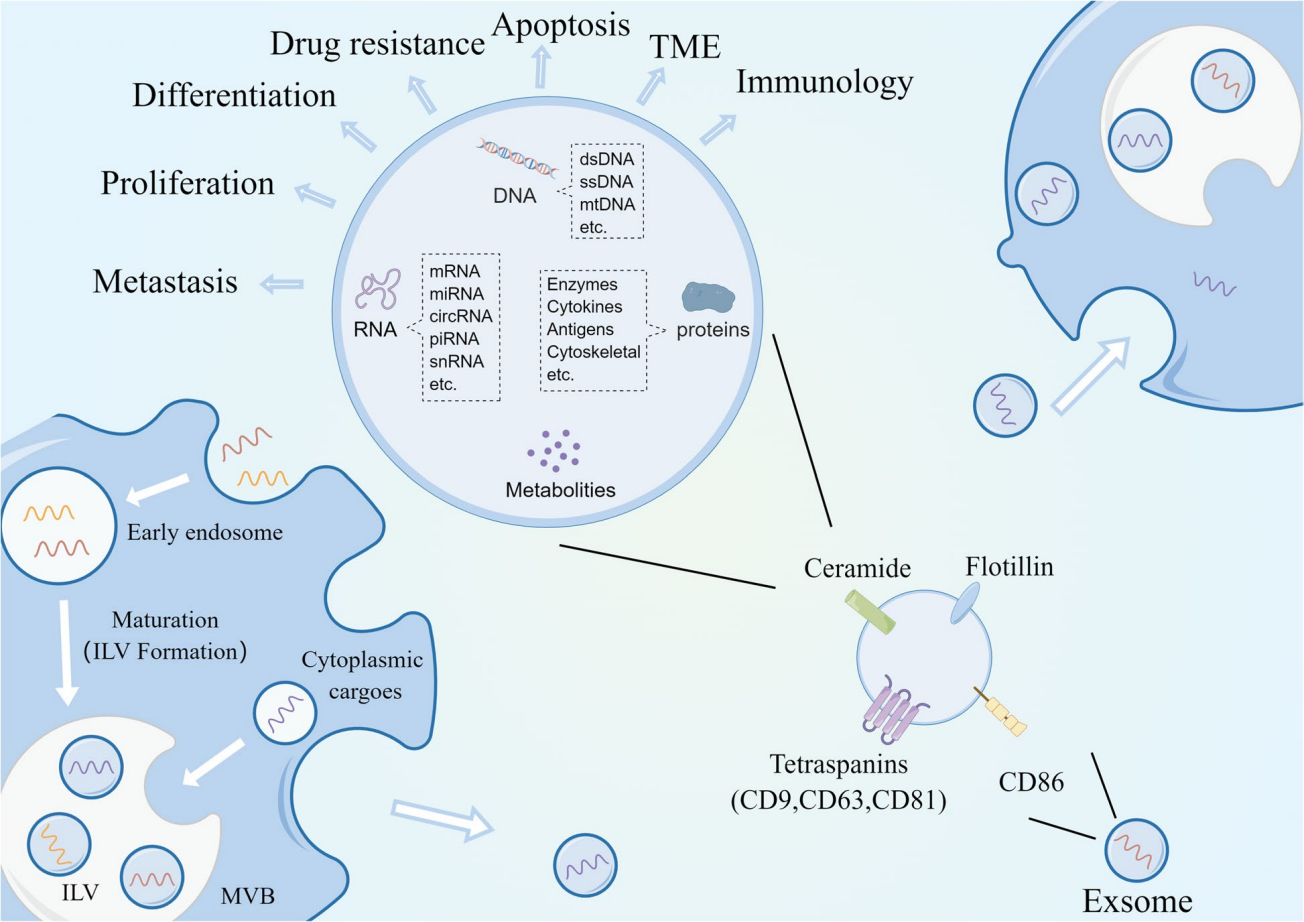
Exosomal biogenesis and regulatory mechanisms. The cell membrane wraps specific cellular material inward to form EEs which mature into MVBs. Meanwhile, MVBs also receive specific cargo from the cytoplasm. Different materials within the MVBs are separated by the membrane to form ILVs. Low-cholesterol MVBs are degraded, while high-cholesterol MVBs fuse with the cell membrane to release ILVs into the circulation. The released ILVs are called exosomes. The exosomes are enriched with cell surface proteins on the membranes such as Tetraspanins (CD9, CD81, CD63), CD86, integrins and ceramide, which are used as exosome markers and recognized by target cells. It is reported that exosomes regulate tumor differentiation, proliferation, apoptosis, EMT, drug resistance, and the TME by transmitting intercellular messages

Exosomes are EVs that are enriched with a variety of substances, including DNA, miRNA, mRNA, cellular metabolites, and proteins [129, 130], and are widely distributed in blood, urine, and saliva [131, 132]. Exosomes are secreted by multiple cells and regulate intercellular communication by delivering specific molecules to the receptor cells [133–135]. Several previous studies have demonstrated that exosomes influence RCC progression, including proliferation, metastasis, regulation of the TME, and drug resistance [136–139]. Exosomes originate from intraluminal vesicles (ILVs) released from multivesicular bodies (MVBs). Briefly, cell membranes sprout inward and wrap specific cellular components to form early endosomes (EE), which then mature into MVBs enriched with ILVs. The MVBs also receive cargo from the cytoplasm. MVBs with low cholesterol content are degraded, whereas others with high cholesterol content are transported to the cell membrane, where they fuse with the cell membrane and release ILVs, which are known as exosomes [140, 141]. These exosomes are enriched with cell surface proteins on the membranes such as Tetraspanins (CD9, CD81, CD63), CD86, integrins and ceramide, which are used as exosome markers and recognized by target cells (Fig. 4) [142, 143]. In recent years, exosomes have become a focal point in cancer research and are widely used in liquid biopsies of various cancers [14, 144–146].

A variety of methods are currently used for isolating exosomes, with ultracentrifugation predominating, including differential centrifugation, density gradient ultracentrifugation, and other ultracentrifugation-based methods [147]. Differential centrifugation is the most commonly used separation method because of its simplicity and efficiency. However, differential centrifugation cannot effectively distinguish other impurities, such as cellular debris, proteins, or other EVs, including cellular microvesicles and apoptotic vesicles, resulting in lower product purity [148]. Density gradient differential centrifugation separates impurities by adding media of similar density to the separated material and extending the centrifugation time [149, 150], which can significantly improve the purity of the separated products. However, this also means that it is more time-consuming and difficult to operate, and prolonged centrifugation may cause structural damage [151]. Immunoaffinity-based methods to isolate exosomes are also very prevalent [152], and a number of commercial reagents or methods have been developed, such as the exoEasy Maxi Kit (QIAGEN, USA), total exosome isolation reagent, and EpCAM isolation beads (Invitrogen, USA). By using antibodies or antibody-characterized magnetic beads, higher-purity exosomes can be captured and purified; however, limitations such as low capture rate and high cost still exist [153]. In recent years, as exosome research has intensified, an increasing number of methods have emerged, including ultrafiltration, membrane affinity chromatography, and size exclusion chromatography; many studies have compared the associated advantages and disadvantages [154–156]. Based on the progression of the above methods, many kits have been developed and are commercially available, such as exoQuick (Qiagen, Netherlands), exoEasy (System Biosciences, USA), Total exosome isolation reagent (Invitrogen, USA), etc. These kits make the extraction and enrichment of exosomes less demanding in equipment, and enable the extraction of larger scale exosomes in less time, which make it more feasible to extend exosome detection to clinic. The extraction efficiency and accuracy of various kits are inconsistent due to differences in their extracting methods and techniques, and several studies have tested and compared them [157–159]. Three key issues still limit the wider clinical application of exosomes: first, how to improve the capture rate of exosomes, second, how to make exosome isolation more cost-effective, and third, how to ensure the purity of exosomes.

### Clinical application of liquid biopsy in RCC

In recent years, with the continuous development of detection techniques for liquid biopsy components, an increasing number of clinical trials are being conducted to apply this new non-invasive test in various clinical practices for RCC. Depending on the purpose, we divided the trials into three categories: diagnostic (including early diagnosis and differential diagnosis), prognostic, and therapeutic. In the following section, we review the clinical trials on liquid biopsy for RCC in the last 5 years.

### Diagnosis of RCC

Early diagnosis of cancer can provide more options for treatment strategies for patients, thus improving patient survival and reducing the consumption of healthcare resources. In addition, identifying cancer from benign tumors and distinguishing between different types of malignant tumors are also crucial for disease control [160]. Therefore, many studies have attempted to use liquid biopsy as a routine method for the clinical diagnosis of RCC (Table 1). As CTCs have long been considered the culprit of tumor metastasis and recurrence, their significance in tumor diagnosis is very limited compared to other components. Only one study in the last 5 years was reported to differentiate between responding and progressing Patients with RCC based on the cytokeratin expression level of CTCs [24]. Multiple mutations were found for cfDNA/ctDNA in the plasma. In 2020, a study detected mutations in 17 of 51 patients with metastatic RCC (mRCC). Among them, the most frequently mutated gene was VHL with a frequency of 41%, followed by BRCA1-associated protein 1 (BAP1) (39%) and recombinant polybromo 1 (PBRM1) (17%) [49]. These ctDNA genetic mutations in the plasma were consistent with the DNA mutations in the corresponding tissues, with a concordance of 77%. However, a study by Sumiyoshi et al. reported that 13 VHL variants were found in 12 of 56 patients with ccRCC (21.6%) with a median variant frequency of 0.78%, while only eight out of 28 patients (28.6%) had plasma VHL variants with VHL mutations in tumor tissue. Owing to its low detection rate, the authors concluded that the analysis of single cfDNA/ctDNA is not applicable to liquid biopsies [48]. In contrast, DNA methylation analysis may be more accurate in patients with RCC, as proposed by two studies conducted in 2020. Lasseter et al. first identified 21 candidate variants in 11 out of 40 patients with mRCC (28%). Based on the methylation levels of 21 cfDNAs, the team constructed methylation scores for the diagnosis of a cohort of 72 individuals (38 Patients with RCC, 34 healthy controls). The results showed that all 34 patients were successfully probed (sensitivity, 100%; specificity, 88%) [61]. Another study further identified 300 differentially methylated regions in plasma cfDNAs and constructed methylation scoring models for diagnosis. According to the scoring model, 67 of 69 Patients with RCC (97.1%) were accurately diagnosed with an area under the receiver operating characteristic curve of 0.990 [60]. Interestingly, the diagnostic model was also applied to urinary cfDNA, with an area under the curve (AUC) of 0.858. Furthermore, the model showed significant differential diagnostic ability. In addition to specific cfDNA analysis, the overall plasma cfDNA level has diagnostic value. According to Yamamoto et al., the AUC of cfDNA levels for detecting RCC was 0.762 (sensitivity, 63.0%; specificity, 78.1%) [41].

**Table 1:** Diagnostic application of liquid biopsy in recent 5 years

	Region	Year	Sample	Detection Method	Cohorts	Detected Abnormality	Practice in clinical	Result	Ref
CTC	USA	2021	Peripheral blood	VERSA Platform, Immunofluorescence	29 RCC patients	CK ( +) CTC counts	Distinguishing progressing and responding patients	AUC 0.79, Sensitivity 73% and Specificity 100%	[24]
ctDNA/ cfDNA	Japan	2021	Plasma	NGS dPCR	56 ccRCC patients 31 healthy control	VHL	Detection of RCC patients	13 VHL mutations were found in 12 of 56 ccRCC patients (21.6%) with median variant frequency of 0.78% VHL cfDNA mutations were found in 8 of 28 patients (28.6%) with VHL tumor DNA mutations Patients with VHL cfDNA mutations tended to show a worse OS	[48]
	USA	2020	Plasma Urine	cfMeDIP–seq NGS	99 RCC patients 28 healthy controls 15 UBC patients	300 DMRs	Detection of RCC patients	67/69 RCC samples (97.1%) were of a higher median methylation score than all control samples with a mean AUC of 0.990 Same analyses were carried out to urine cfDNA from patients with RCC and healthy controls, with the mean AUC of 0.858	[60]
							Distinguishing RCC and UBC	Using methylation score to compare patients with RCC and UBC, resulting in a mean AUC of 0.979	
	USA	2020	Plasma	cfMeDIP–seq Target sequencing	Cohort 1: 40 mRCC patients Cohort 2: 38 RCC patients 34 healthy controls	Methylation level of 21 cfDNA variants	Detection of mRCC patients	cfDNA variant analysis via targeted sequencing detected 21 candidate variants in 11 of 40 mRCC patients (28%), which can improve the sensitivity combined with tumor DNA variant analysis All of 34 mRCC patients are detected through cfMeDIP–seq (sensitivity 100%, specificity 88%), compared with that cfDNA variant analysis identified variants in 7 patients (21%)	[61]
	Canada	2020	Plasma	Target sequencing	55 mRCC patients	VHL, BAP1, PBRM1 et al	Detection of mRCC patients	17 of 51 mRCC patients detected cfDNA variants. The most frequent mutated genes are VHL, BAP1 and PBRM1 (the frequency is 41%, 39%, 17%, respectively). The concordance of mutated genes profiling between cfDNA variants in plasma and tumor DNA variants in matched tissues is 77%	[49]
	Japan	2018	Plasma	qPCR Microfluidics-based platform	92 RCC patients 41 healthy controls	Plasma cfDNA level	Detection of RCC patients	AUC 0.762, Sensitivity 63.0% and Specificity 78.1%	[41]
cfRNA	Portugal	2022	Plasma	ddPCR	124 RCC patients 15 oncocytomas patients 64 healthy controls	miR-21-5p miR-155-5p	Detection of RCC patients	Sensitivity 89.52%, specificity 54.69% and accuracy 77.66%	[161]
					124 RCC patients	miR-21-5p miR-155-5p	Detection of early stages RCC	Sensitivity 92.42%, specificity 34.38% and accuracy 63.85%	
					124 RCC patients 15 oncocytomas patients	miR-126-3p miR-200b-3p	Distinguishing ccRCC and other RCC subtypes	Sensitivity 80.46%, specificity 56.76% and accuracy 73.39%	
	Canada	2021	Urine	qPCR	76 ccRCC patients 8 benign renal tumor patients 16 healthy contrls"	Circ-EGLN3 Circ-SOD2	Detection of RCC patients	69% of samples detected urinary circEGLN3 and 60% of samples detected urinary circACAD11 circEGLN3 levels were significantly different between the healthy controls versus ccRCC patients (*P* < 0.05) The AUC of circEGLN3 and circSOD2 was of 0.71 and 0.68, respectively, for distinguishing cancer patients versus non-neoplastic patients Urinary circEGLN3 level of ccRCC patients was lower than that of healthy controls, while tissue circEGLN3 level was higher of ccRCC patients	[174]
	China	2021	Serum	qPCR	123 RCC patients 118 healthy controls	miR-21-5p miR-150-5p miR-145-5p miR-146a-5p	Detection of RCC patients	AUC 0.938, sensitivity 90.79%, specificity 93.75%	[162]
	China	2020	Serum	qPCR	113 RCC patients 79 healthy controls	LncRNA-C00886	Detection of RCC patients	AUC 0.803, sensitivity 67.09%, specificity 89.87%	[172]
							Detection of early stages RCC patients	AUC 0.800, sensitivity 71.05%, specificity 89.87%	
							Detection of non-metastasis RCC patients	AUC 0.830, sensitivity 73.33%, specificity 89.87%	
	Portugal	2020	Urine	qMSP	Cohort 2: 38 ccRCC patients 15 metastasis ccRCC patients 57 healthy controls Cohort 3: 171 ccRCC patients 85 healthy controls	Methylation level of miR-30a-5p	Detection of ccRCC patients Detection of metastasis ccRCC patients	Cohort 2: AUC 0.6873, sensitivity 83%, specificity 53% Cohort 3: AUC 0.6702, sensitivity 63%, specificity 67% Cohort 2: AUC 0.7684, sensitivity 80%, specificity 71%	[79]
	China	2020	Serum	qPCR	146 RCC patients 150 healthy controls	miR-224-5p miR-34b-3p miR-182-5p	Detection of RCC patients	AUC 0.855, sensitivity80.3%, specificity66.3%	[163]
	China	2020	Serum	qPCR	Testing cohort: 70 RCC patients 70 healthy controls Validating cohort: 40 RCC patients 40 healthy controls	miR-20b-5p, miR-30a-5p, miR-196a-5p	Detection of RCC patients	Testing cohort: AUC 0.949, sensitivity 92.8%, specificity 80.0% Validating cohort: AUC 0.938, sensitivity 92.5%, specificity 80.0%	[164]
	Canada	2020	Urine	qPCR	30 oncocytomas patients 26 progressive ccRCC-SRM patients 24 non-progressive ccRCC-SRM patients	9 miRNAs miR-328-3p	Distinguishing RCC- SRM and oncocytoma Detection of ccRCC patients	9 urinary miRNAs were differentially expressed between renal oncocytoma (≤ 4 cm) and ccRCC-SRMs (pT1a; ≤ 4 cm), where miR-432-5p and miR-532-5p showed the most measurable discriminatory ability (AUC 0.71, AUC 0.70, respectively) miR-328-3p was significantly down-regulated in progressive ccRCC-SRMs and showed significant discriminatory ability (AUC: 0.68)	[79]
	China	2018	Serum	qPCR	46 RCC patients 46 healthy controls	LncRNA-GIHCG	Detection of RCC patients	AUC 0.920, sensitivity 87%, specificity 84.8%	[173]
							Detection of early stage RCC patients	AUC 0.886, sensitivity 80.7%, specificity 84.8%	
	Ukraine	2018	Urine	qPCR	52 RCC patients 15 oncocytoma patients 15 healthy controls	miR-15a	Distinguishing RCC and benign renal tumor	AUC 0.955, sensitivity 100%, specificity 98.1%	[169]
	Germany	2018	Serum	qPCR	86 ccRCC patients 55 benign renal tumor patients 28 healthy controls	miR-122-5p miR-206	Detection of ccRCC patients	AUC 0.733, sensitivity 57.1%, specificity 83.8%	[165]
Protein	India	2021	Serum	Elisa	60 RCC patients 60 non-tumor controls	GRP78	Detection of RCC patients	AUC 0.739, sensitivity 71.7%, specificity 66.7%	[179]
	USA	2021	Plasma	Elisa	143 mRCC patients 137 18–25 years old healthy controls 252 50–80 years old healthy controls	hPG80	Detection of mRCC patients	Compared to 18–25 years old healthy group: AUC 0.93, accuracy 0.89 Compared to 50–80 years old healthy group: AUC 0.84, accuracy 0.77	[180]
	Canada	2020	Urine	LC–MS/MS	27 oncocytoma (≤ 4 cm) patients 23 progressive ccRCC-SRM patients 21 non-progressive ccRCC-SRM patients 20 healthy controls	GLRx、CST3、SLC9A3R1、HSPE1、FKBP1a、EEF1G et al	Detection of early-stage ccRCC patients	GLRx (AUC = 0.72, *P* = 0.0047) showed the most significant discriminatory ability between ccRCC-SRM and healthy controls, followed by SLC9A3R1 (AUC = 0.70), HSPE1 (AUC = 0.70), FKBP1A (AUC = 0.65) and EEF1G (AUC = 0.65) (*P* < 0.05) Diagnostic model based on the expression of 7 proteins (DDT, EEF1G, EPB41L3, HSPE1, MUC4, RAP1B and SLC9A3R1) showed the most significant discriminatory ability (AUC: 0.82), outperforming all single protein markers	[178]
							Distinguishing renal oncocytoma (≤ 4 cm) and early-stage ccRCC	C12orf49 (AUC = 0.77, *P* = 0.0001) showed the most significant discriminatory ability between ccRCC-SRM and renal oncocytoma, followed by EHD4 (AUC = 0.64, *p* = 0.049) Diagnostic model based on the expression of 3 proteins (C12orf49、EHD4 and PPA1) showed the most significant discriminatory ability (AUC: 0.85), outperforming all single protein markers	
							Distinguishing progressive and non-progressive early-stage ccRCC	EPS8L2 (AUC = 0.76, *p* = 0.0037) showed the most significant discriminatory ability between progressive and non-progressive ccRCC-SRM, followed by CHMP2A (AUC = 0.70, *p* = 0.034), PDCD6IP (AUC = 0.68), CNDP2 (AUC = 0.63) and CEACAM1 (AUC = 0.66)(*P* < 0.05) Diagnostic model based on the expression of 2 proteins (EPS8L2 and CCT6A) showed the most significant discriminatory ability (AUC = 0.81), outperforming all single protein markers	
	USA	2019	Urine	Plasmonic biosensor	20 RCC patients 20 healthy controls 8 BLCA patients 10 diabetic nephropathy patients	PLIN-2	Detection of RCC patients	Median urine PLIN-2 concentrations in ccRCC patients (43 ng/mL) were significantly higher (*P* < 0.001) than healthy groups (0.3 ng/mL), BLCA patients (0.5 ng/mL) and diabetic nephropathy patients (0.6 ng/mL)	[128]
Metabolites	Portugal	2021	Urine	HS–SPME–GC–MS	75 ccRCC patients 75 health control	6 volatiles metabolites	Detection of RCC patients	The diagnostic model was consisted of 6 volatile metabolites The diagnostic ability of ccRCC patients: AUC 0.869, sensitivity 83%, specificity 79%, accuracy 79% The diagnostic ability of stage I ccRCC patients: AUC 0.799, sensitivity 84%, specificity 73%, accuracy 76% The diagnostic ability of stage III-IV ccRCC patients: AUC 0.911, sensitivity 83%, specificity 84%, accuracy 84%	[101]
	Italy	2021	Urine	GC/MS Gas sensor array	40 ccRCC patients 8 healthy controls	8 volatiles metabolites	Detection of ccRCC patients	8 volatile metabolites was differentially expressed in at least 70% ccRCC patients and consisted as a diagnostic model Analyzed through GC/MS, the diagnostic ability of the model: AUC 0.979, sensitivity 85.7%, specificity 100%, accuracy 92.9% (training cohort); AUC 0.875, sensitivity 83.3%, specificity 100%, accuracy 91.7% (testing cohort) Analyzed through Gas Sensor Array, the diagnostic ability of the model: AUC 0.979, sensitivity 100%, specificity 85.7%, accuracy 92.9% (training cohort); AUC 0.906, sensitivity 100%, specificity 83.3%, accuracy 91.7% (testing cohort)	[102]
	Germany	2021	Urine	LC–MS NMR	41 early stage RCC patients 29 advanced stage RCC patients	16 urinary metabolites	Distinguishing early and advanced stage RCC patients	A model consisting of 16 metabolites was used for distinguishing early and advanced stage RCC patients: AUC 0.95, sensitivity 80%, specificity 91%, accuracy 86%	[105]
	China	2020	Urine	LC–MS	39 RCC patients 22 benign renal tumor patients 68 healthy controls	6 urinary metabolites	Distinguishing RCC and benign renal tumor	A model consisting of 3 metabolites (cortolone, testosterone and l-2-aminoadipate adenylate) was used for benign and malignant renal tumor distinction: AUC 0.868, sensitivity 75%, specificity 100% (tenfold cross-validation of testing cohort)	[175]
							Detection of RCC patients	A model consisting of 3 metabolites (aminoadipic acid, 2-(formamido)-N1-(5-phospho-d-ribosyl) acetamidine and alpha-N-phenylacetyl-l-glutamine) was used for detection RCC patients: AUC 0.841, sensitivity 75%, specificity 88.6% (tenfold cross-validation of testing cohort)	
	Japan	2020	Urine	LC–MS	69 stage I-II RCC patients 18 stage III-IV RCC patients 60 benign renal tumor patients	9 urinary metabolites	Distinguishing RCC and benign renal tumor	A model consisting of 5 metabolites (L-glutamic acid, lactate, D-sedoheptulose 7-phosphate, 2-hy-droxyglutarate and myoinositol) was used for detection RCC patients: AUC 0.966, sensitivity 93.1%, specificity 95%	[176]
	Poland	2020	Urine	AuNPET LDI MS	50 RCC patients 50 healthy controls	15 urinary metabolites	Detection of RCC patients	15 urinary metabolites were identified abnormal distribution in RCC patients' urine (7 upregulation and 8 downregulation), where 3,5-Dihydroxyphenylvaleric acid showed the most significant diagnostic value (AUC 0.844) A model consisting of all 15 metabolites was used for detecting RCC patients: AUC 0.915, efficiency 88%, efficiency 86%	[103]
	China	2019	Urine	UPLC-MS	146 BLCA patients 115 RCC patients 142 healthy controls	16 urinary metabolites	Detection of RCC patients	A model consisting of 6 metabolites (α-CEHC, β-cortolone, deoxyinosine, flunisolide, 11b,17a,21-trihydroxypreg-nenolone and glycerol tripropanoate) was used for distinguishing cancer patients from healthy controls: AUC 0.950 (discovering group); AUC 0.867 (external validating group)	[104]
							Distinguishing BLCA and RCC patients without hematuria	A model consisting of 6 metabolites (4-ethoxymethylphenol, prostaglandin F2b, thromboxane B3, hydroxybutyrylcarnitine, 3-hydroxyphloretin and N'-formylkynurenine) was used for distinguishing BLCA and RCC patients without hematuria: AUC 0.829 in discovering group; AUC 0.76 in external validating group	
							Distinguishing BLCA and RCC patients with hematuria	A model consisting of 4 metabolites (1-hydroxy-2-oxopropyl tetrahydropterin, 1-acetoxy-2-hydroxy-16-heptadecyn-4-one, 1,2dehydrosalsolinol and L-tyrosine) was used for distinguishing BLCA and RCC patients with hematuria: AUC 0.913 (discovering group)	
	China	2019	Urine	LC–MS	100 RCC patients 34 benign renal tumor patients 129 healthy controls	18 urinary metabolites	Detection of RCC patients	A model consisting of 9 metabolites (N-Jasmonoyltyrosine, Tetrahydroaldosterone-3-glucuronide, Androstenedione, Dopamine 4-sulfate, 3-Methylazelaic acid, Cortolone-3-glucuronide, 7alpha-hydroxy-3-oxochol-4-en-24-oic Acid, Cortolone-3-glucuronide and Lithocholyltaurine) was used for distinguishing cancer patients from healthy controls: AUC 0.905 (testing cohort); AUC 0.885 (validating cohort) N'-formylkynurenine showed a significant discriminating ability of detecting RCC patients (AUC 0.808, sensitivity 84.8%, specificity 83.8%)	[177]
							Distinguishing RCC and benign renal tumor	A model consisting of 3 metabolites (L-3-hydroxykynurenine, 1,7-dimethylguanosine and tetrahydroaldosterone-3-glucuronide) was used for distinguishing RCC patients from benign renal tumor patients: AUC 0.834 in testing cohort; AUC 0.816 for tenfold cross-validation	
							Distinguishing early and late stages of RCC	A model consisting of 5 metabolites (thymidine, cholic acid glucuronide, alanyl-proline, isoleucyl-hydroxyproline, and myristic acid) was used for distinguishing early (stage I-II) from late stages (stage III-IV) of RCC: AUC 0.881 in testing cohort; AUC 0.813 for tenfold cross-validation	
Exosome	Spain	2021	Plasma	Differential ultracentrifugation, qPCR, NGS, dPCR	13 RCC patients 15 healthy controls	Exosomal mtDNA VH1, CγB	Detection of RCC patients	dPCR and qPCR demonstrated that VH1 and CγB were of a significant discrimination ability for RCC and healthy group (F phase) VH1: AUC = 0.825, *P* < 0.0001 for VH1-short; AUC = 0.833, *P* < 0.0001 for VH1-long CγB: AUC = 0.755, *P* < 0.0001 for CγB-short; AUC = 0.810, *P* < 0.0001 for CγB-long	[183]
	China	2020	Plasma	exoEasy maxi kit, qPCR	22 RCC patients 16 healthy controls	Exosomal miRNA miR-92a-1-5p, miR-149-3p, miR-424-3p	Detection of RCC patients	Compared with healthy controls, the levels of exsomal miR-149-3p and miR-424-3p were significantly up-regulated, while miR-92a-1-5p was down-regulated miR-149-3p: AUC 0.7188, sensitivity 75.0%, specificity 72.7% miR-424-3p: AUC 0.7727, sensitivity 75.0%, specificity 81.8% miR-149-3p: AUC 0.8324, sensitivity 87.5%, specificity 77.3%	[181]
	China	2019	Urine	Differential ultracentrifugation, Agilent 2100 Bioanalyzer, NGS	70 early‐stage ccRCC patients 30 early‐stage PC patients 30 early‐stage BLCA patients 30 healthy controls	Exosomal miRNA miR-30c-5p	Detection of ccRCC patients	Exosomal miRNA miR-30c-5p levels in ccRCC patients' urine were significantly lower than those in healthy controls, where was no significant differences between BLCA cancers, PC cancers and healthy controls The diagnostic value of exosomal miR-30c-5p for ccRCC patients: AUC 0.819, sensitivity 68.57%, specificity 100%	[182]
	China	2018	Serum	Total exosome isolation reagent, EpCAM isolation beads, Flow cytometry	82 ccRCC patients received nephrectomy 80 healthy controls	Exosomal miRNA miR-210, miR-1233	Detection of RCC patients	Exosomal miRNA miR-210 and miR-1233 levels in ccRCC patients' serum were significantly lower than those in healthy controls miR-210: AUC 0.69, sensitivity 70%, specificity 62.2% miR-1233: AUC 0.82, sensitivity 81%, specificity 76%	[153]

Urine samples have been used more often in clinical trials of cfRNA than cfDNA/ctDNA. Most trials constructed diagnostic panels consisting of several cfRNAs to detect Patients with RCC [161–165]. Some of these diagnostic panels had excellent diagnostic ability; for example, panels composed of miR-21-5p, miR-150-5p, miR-145-5p, and miR-146a-5p had an AUC of 0.938 (sensitivity, 90.79%; specificity, 93.75%) [162]. Interestingly, miR-21-5p was also included in a diagnostic panel in another clinical trial, which was used to differentiate between benign and malignant tumors and to probe Patients with RCC at early stages [161]. miR-21-5p has been reported to play an important role in various cancers and may be associated with mesenchymal–epithelial transition, suggesting its feasibility for clinical liquid biopsies [166–168]. Moreover, several miRNA panels have differential diagnostic value. Among them, miR-126-3p was able to distinguish ccRCC from other pathological types of RCC with an accuracy of 71.77% (sensitivity, 78.16%; specificity, 56.76%), and the accuracy of the combination of miR-126-3p and miR-200-3p increased to 73.39% (sensitivity, 80.46%; specificity 56.76%) [161]. Single miRNAs are more efficient and economical than panels comprising multiple miRNAs. Yulian Mytsyk et al. showed that miR-15a had significant differential diagnostic value for benign tumors and RCC, with an AUC of 0.955 (sensitivity, 100%; specificity, 98.1%) [169]. However, the sample size in this trial was small, and a larger cohort is needed to test its reliability. Another study identified nine urinary miRNAs that were differentially expressed between small renal cancer masses and eosinophilia, a benign renal tumor, where miR-432-5p and miR-532-5p had the most significant discriminatory value, with AUCs of 0.71 and 0.70, respectively [170]. In addition to cell-free miRNA levels, miRNA methylation level is also a potential diagnostic indicator. The methylation level of miR-30a-5p in urine can be measured to detect RCC and identify patients with mRCC with an AUC of 0.855 (sensitivity, 80.3%; specificity, 66.3%) [79]. Previous studies have demonstrated that miR-30a-5p inhibits cancer progression by regulating GRP78 and zinc finger E-box binding homeobox [171, 172], which has the potential to be a biomarker in liquid biopsies and has been used in diagnostic panels in recent studies [164]. In addition to miRNAs, two lncRNAs are also used as diagnostic markers, namely lncRNA-GIHCG and lncRNA-C00886, with AUCs of 0.920 and 0.803, respectively [173, 174]. Among these, lncRNA-GIHCG was found to be significantly decreased post-surgery and has potential in the monitoring of surgical outcomes. Differential expression of circRNA has also been detected in urine and used as a diagnostic indicator; however, it faces three problems: first, the detection rate is insufficient in Patients with RCC; second, the AUC is low compared to other RNA species; and third, the differential expression of serum circRNA is inconsistent with tissue circRNA [175]. Metabolomic changes are mainly reflected in urine, and several clinical trials have constructed diagnostic models to detect Patients with RCC by identifying differentially expressed metabolites therein [101–105, 176–178]. Based on the differential expression of metabolites, several studies have assembled large and complex diagnostic panels with significant diagnostic value and a very high AUC [103, 105, 178]. However, these studies were not without their shortcomings; overly complex diagnostic models are difficult to scale up to clinical application and are weakly reproducible, comprising strong inconsistencies across patients. Sato et al. used five differentially expressed metabolites to detect ccRCC in a cohort of 87 patients with ccRCC and 60 patients with benign urologic disease, with an AUC of 0.966 (sensitivity, 93.1%; specificity, 95%) [177]. Similarly, Zhan Wang et al. performed diagnostic analysis using a panel of six metabolites, resulting in an AUC of 0.950 in the test group and 0.867 in the validation group [104]. This trial further explored the ability to distinguish between bladder cancer and RCC. By using hematuria to classify patients, the trial improved the accuracy of the results and reduced the complexity of the diagnostic panel. The method of classifying patients according to their symptoms may be a useful way to avoid complex diagnostic panels.

Metabolomics can reflect altered pathways in RCC as well as altered renal function, which could contribute to further revealing the pathogenesis and explaining clinical symptoms, such as the relationship between tryptophan and immune regulation [178]. However, related studies have explored this topic to a limited extent and more in-depth analyses are needed. Unlike metabolomic analysis, protein analysis typically uses a single protein to diagnose patients. Only one study used multiple proteins to probe patients with early stage RCC and to discriminate between benign and malignant small renal masses [179]. Although its diagnostic value is not as remarkable as that of metabolomics-based approaches, single biomarker analysis significantly improves its feasibility for clinical application. Proteins used in the last 5 years of research include GRP78, human circulating progastrin 80 (hPG80), and perilipin-2 (PLIN-2) [128, 180, 181]. Among them, probing patients with mRCC based on circulating hPG80 levels showed the most significant diagnostic value [181].

With the increasing understanding of exosome oncogenesis and the continuous development of isolation techniques, the diagnostic value of exosomes is becoming an important tool for cancer diagnosis. In the last 5 years, several studies have extracted exosomal nucleic acids, including miRNA and mtDNA, to diagnose patients with RCC. In contrast to cfRNAs, exosomes typically prefer to use a single miRNA as a biomarker rather than a panel [153, 182, 183]. Multiple exosomal miRNAs have shown significant diagnostic value, including miR-149-3p (AUC, 0.8324; sensitivity, 87.5%; specificity, 77.3%), miR-30c-5p (AUC, 0.819; sensitivity, 68.57%; specificity, 100%), and miR-1233 (AUC, 0.82; sensitivity, 81%; specificity, 76%). Notably, miR-15a is different as a cfRNA between Patients with RCC and a healthy population, but not as an exosomal miRNA [153]. Moreover, Arance et al. demonstrated the potential of mtDNA for use in clinical practice [184]. Notably, this study discussed the difference in the purity of mtDNA in different phases of differential centrifugation to facilitate subsequent analysis, but further work with a larger sample size is needed.

### Prognosis of RCC

Apart from its diagnostic value, liquid biopsy can also predict patients’ grades, stages, and survival to identify high-risk patients and predict their risk of metastasis and recurrence (Table 2). CTCs and cfDNA/ctDNA are correlated with overall survival (OS) or progression-free survival (PFS). Basso et al. supposed that patients with CTCs above 3 had a significantly shorter OS (13 vs. 52.8 months) and PFS (5.8 vs. 15 months) than patients with CTCs below 3 [185]. Moreover, both studies demonstrated that shorter cfDNA fragments were remarkably associated with shorter PFS (*P* = 0.004 and *P* = 0.006, respectively) [41, 50]. Notably, cfDNA mutation status, fragment size, and the proportion of cfDNA fragments (PCF) were associated with prognosis in the mRCC patient group (*P* = 0.010, *P* = 0.011, and *P* = 0.007, respectively) but not in the metastasis-free Patients with RCC group [50]. Additionally, cfRNA and protein levels were used to assess patient survival. Serum miR-122-5p and miR-206 levels (all *P* < 0.005) and urinary miR-328-3p levels (hazard ratio (HR) = 0.29, *P* = 0.042) were also significantly associated with OS [165, 170]. Among them, serum levels of miR-122-5p and miR-206 were also associated with the grading, staging, and distant metastasis of ccRCC [165]. Kohli et al. showed that patients with plasma hPG80 levels above 4.5 pM had significantly shorter OS than the rest of the patients (12 months vs. 31.2 months; *P* = 0.0031) [181]. Adding the index to the IMDC prognostic scores improved risk prediction ability. Gigja Gudbrandsdottir et al. reported that the chemokines IL-27 and IL-6, and gp30, the receptor for IL-6, have predictive abilities for survival time in patients with RCC. However, the predictive abilities vary in tumors of different sizes [117]. In addition, urinary NGAL levels implicated a predicting ability of risk of death (HR = 5.5, *P* = 0.005), where its HR for cancer-specific death in patients with levels above 2.19 ng/mmol was 7.2 (*P* = 0.001) [186].

**Table 2:** Prognostic application of liquid biopsy in recent 5 years

	Region	Year	Detected Abnormality	Sample	Detection Method	Cohorts	Practice in clinical	Result	Ref
CTC	Italy	2021	Enumeration	Peripheral blood	CellSearch System	195 metastasis RCC patients	Predicting patients' survival	Patients with at least 3 CTCs had a shorter OS of 13.8 months versus 52.8 months in those with fewer than 3 CTCs Patients had at least 3 CTCs, with a median PFS of 5.8 versus 15 months in the remaining patients	[184]
	China	2019	Beclin-1	Peripheral blood	Can Patrol CTC enrichment technique, RNA ISH	58 metastasis-free RCC patients 11 metastasis RCC patients	Predicting patients' metastasis	The number of preoperative Beclin1-positive CTCs in the metastatic group was significantly higher than the number of Beclin1-negative CTCs (*P* < 0.05) The difference between the number of Beclin1-positive CTCs and Beclin1-negative CTCs in the metastasis-free group was not statistically significant (*P* > 0.05)	[25]
ctDNA/ cfDNA	Japan	2019	Status and fragment size of cfDNA or ctDNA	Plasma	NGS ddPCR	53 RCC patients	Predicting patients' survivals	ctDNA status was associated with PFS and CSS (*P* = 0.061, *P* < 0.01, respectively) cfDNA fragment size was significantly associated with PFS and CSS (*p* = 0.004, *p* = 0.011, respectively)	[50]
	Japan	2018	Plasma cfDNA fragment size	Plasma	qPCR Microfluidics-based platform	92 RCC patients 41 healthy controls	Predicting patients' survivals	Shorter cfDNA fragment size was negatively associated with progression-free survival (*p* = 0.006)	[41]
cfRNA	Canada	2020	miR-328-3p	Urine	qPCR	30 oncocytomas patients 26 progressive ccRCC-SRM patients 24 non-progressive ccRCC-SRM patients	Predicting patients' survivals	Patients with high miR-328-3p expression levels had significantly longer OS (HR = 0.29, *p* = 0.042) compared to patients with low miR-328-3p expression levels	[79]
	Germany	2018	miR-122-5p miR-206	Serum	qPCR	86 ccRCC patients 55 benign renal tumor patients 28 healthy controls	Predicting patients' grade and metastasis	miR-122-5p levels were significantly increased in metastasized ccRCCs (cM0 vs. cM1; *p* = 0.045) and advanced Fuhrman Grade (G1/2 vs. G3/4; *p* = 0.001) Serum miR-206 expression was significantly increased in advanced pT-stage (pT1/2 vs. pT3/4; *p* = 0.006) and metastasized ccRCC (cM0 vs. cM1; *p* = 0.002)	[165]
							Predicting patients' survivals	Univariate Cox Regression analysis showed that elevated miR-122-5p and miR-206 serum levels were correlated with a shorter period of progression-free, cancer-specific, and overall survival (all *P* < 0.005)	
Protein	India	2021	GRP78	Serum	Elisa	60 RCC patients 60 non-tumor controls	Predicting the metastasis of RCC	GRP78 expression was significantly higher in RCC patients with metastatic than in those without metastasis (*P* < 0.001). Predicting ability of metastasis or non-metastasis RCC: AUC 0.954, sensitivity 100%, specificity 90.4%	[179]
							Predicting the grade of RCC	Median level of serum GRP78 increases with higher grade of RCC (*P* < 0.001). Predicting ability of high or low-grade RCC: AUC 0.948, sensitivity 92%, specificity 83%	
	Norway	2021	IL-6, IL-27, IL-31, OSM, CNT-f, IL-6Rα, gp130	Serum	Luminex immune-bead technology and a high-sensitivity kit	159 RCC patients with nephron sparing surgery, a radical nephrectomy or a cyto-reductive nephrectomy	Predicting patients' recurrence	Kaplan–Meier analysis showed IL-27 had a significantly predictive ability of recurrence (*P* = 0.026) Cox multivariate regression consisted of IL-6 and IL-27 showed that IL-6 had a significantly predictive ability of recurrence (*P* = 0.004) but not IL-27 (*P* = 0.082) Kaplan–Meier analysis showed both of IL-27 and IL-6 had significantly predictive abilities of recurrence for ccRCC patients withlarge tumors (diameter > 7.0 cm) (*P* = 0.014, *P* = 0.026, respectively)	[117]
							Predicting patients' survivals	Kaplan–Meier analysis and multivariate regression analysis showed IL-6 had a significantly predictive ability of disease-specific survival (DSS) (*P* < 0.026, *P* = 0.001, respectively) Kaplan–Meier analysis showed IL-6 could predict DSS for patients with a tumor diameter from a 4 to 7 cm and > 7 cm (*P* = 0.001, *P* = 0.02, respectively), and IL-27 could predict DSS for patients with a tumor diameter > 7 cm (*P* = 0.025) Kaplan–Meier analysis showed IL-6 could predict OS for ccRCC patients (*P* = 0.001) but not IL-27 (*P* = 0.066) Kaplan–Meier analysis showed IL-6 could predict DSS for patients with a tumor diameter from a 4 to 7 cm but not > 7 cm (*P* = 0.018, *P* = 0.063, respectively), while gp130 could only predict DSS for patients with a tumor diameter > 7 cm (*P* = 0.001)	
	USA	2021	Hpg80	Plasma	Elisa	89 RCC patients	Refining IMDC prognostic scores	Patients with high hPG80 levels (> 4.5 pM) had a shorter OS than other patients (12 vs. 31.2 months, respectively; *p* = 0.0031) Adding hPG80 levels > 4.5 pM in the IMDC risk scores showed better significance slightly and more refined discriminating ability (*p* = 0.0046)	[180]
	France	2021	SAA2, CFB	Plasma	Elisa	59 mRCC patients with sunitinib or bevacizumab treatment	Predicting the metastasis of RCC	Combination of Fuhrman grade and levels of SAA2 and CFB showed better ability of predicting time to relapse. The model combining Fuhrman grade and CFB showed the best C-Index (C-Index = 0.7273), followed by the three covariates together (C-Index = 0.7163) and Fuhrman grade alone (C-Index = 0.6948)	[186]
	France	2021	NGAL	Urine	ARCHITECT C4100	50 RCC patients	Evaluating post-operative risk of progression and death	ccRCC patients with NGAL level above 2.19 ng/mmol had a 5-time fold higher risk of progression than patients with NGAL level below the threshold (HR = 5.5, *p* = 0.005). The HR of cancer specific death for patients with a level of NGAL above 2.19 ng/mmol was of 7.2 (*p* = 0.001)	[185]
	Italy	2020	PD-1, PD-L1, BTN3A1	Plasma	Elisa	Testing cohort: 21 mccRCC patients with nivolumab treatment Validating cohort: 20 mccRCC patients with nivolumab treatment 15 localized ccRCC patients	Predicting metastasis of ccRCC patients	The mean levels of plasma immune checkpoint levels in metastatic ccRCC patients were significantly higher than localized RCC patients (PD-1: 2.79 vs. 1.54 ng/mL, *p* = 0.003; PD-L1: 0.62 vs. 0.49 ng/mL, *p* = 0.03) Plasma immune checkpoint levels were correlated to number and localization of metastatic sites	[187]
	USA	2019	PLIN-2	Urine	Plasmonic biosensor	20 RCC patients 20 healthy controls 8 BLCA patients 10 diabetic nephropathy patients	Predicting the size of RCC tumor	The urine PLIN-2 concentrations were associated to tumor size which Spearman correlation coefficient is 0.59 (*P* < 0.009)	[128]
Metabolites	Japan	2022	5 urinary metabolites	Urine	LC–MS/MS	56 ccRCC patients 10 benign urological tumor	Predicting the recurrence	A model consisting of 5 metabolites (lactic acid, glycine, 2-HG, succinic acid, and kynurenic acid) showed significant predicting value for ccRCC recurrence (AUC 0.894, sensitivity 88.9%, specificity 88.0%) and T3 ccRCC patients (AUC 0.903, sensitivity 88.9%, and specificity 100%)	[100]
	Japan	2020	9 urinary metabolites	Urine	LC–MS	69 stage I-II RCC patients 18 stage III-IV RCC patients 60 benign renal tumor patients	Predicting the clinical stage	A model consisting of 4 metabolites (L-kynurenine, L-glutamine, fructose 6-phosphate and butyrylcarnitine) was used for predicting III-IV stage of RCC patients: AUC 0.837, sensitivity 88.5%, specificity 75.4%	[176]
Exosome	Spain	2021	Exosome mtDNA VH1, CγB, HBB	Plasma	Differential ultracentrifugation, qPCR, NGS, dPCR	13 RCC patients 15 healthy controls	Predicting the metastasis of RCC	qPCR showed that VH1-short, VH1-long and CγB-short (B phase) were of a significant difference in metastatic group and non-metastatic group (*p* = 0.020, *p* = 0.035, *p* = 0.078, respectively) dPCR showed that VH1-short (B phase) and CγB-short (C phase) were of a significant difference in metastatic group and non-metastatic group (*p* = 0.069, *p* = 0.037, respectively) dPCR and qPCR showed that HBB-long were of a more significant difference in metastatic group and non-metastatic group in most phases	[183]

Furthermore, liquid biopsy can predict the grades, metastasis, and recurrence of RCC. A 2019 study classified CTCs into Beclin 1-positive and Beclin 1-negative CTCs by measuring the cellular levels of Beclin 1 [25]. This trial showed that the number of pre-operative Beclin 1-positive CTCs was significantly higher than that of Beclin 1-negative CTCs in patients in the metastatic group (*P* < 0.05), while there was no significant difference in the non-metastatic group. In addition, protein analysis showed excellent predictive ability for RCC grade and stage. Many proteins, including GRP78, Serum Amyloid A2 (SAA2), Complement Factor B (CFB), circulating cytokines, immune checkpoints in plasma, and PLIN-2 in urine, show measurable differences among patients with different progressions [180, 181, 187, 188]. Briefly, GRP78 can predict patient grades and metastasis [180]; SAA2 and CFB were associated with patient recurrence, where combining Fuhrman grade with CFB levels showed the best C-index to predict recurrence [187]; IL-6 and IL-27 can predict recurrence of RCC, but as with survival prediction their abilities to predict recurrence vary across tumor sizes [117]; plasma immune checkpoints, including PD-1, PD-L1, and BTN3A1, can predict metastasis, which is correlated with the number and location of metastases [188]; and urinary PLIN-2 levels can predict tumor size [186]. Compared to protein, metabolite analysis has been less reported in prognosis prediction, with only two studies in the last 5 years constructing metabolite models to predict patient grades and metastasis [100, 177]. Finally, mtDNA in different phases of exosomes is strongly associated with RCC metastasis. dPCR and qPCR showed that mtDNA HBB-long had the most significant difference between the metastatic and non-metastatic groups in most phases. However, this conclusion needs to be supported by larger patient datasets [184].

### Monitoring and predicting responses of RCC therapy

Surgery is the preferred treatment strategy for patients with RCC owing to its insensitivity to chemotherapy and radiotherapy. In recent years, many adjuvant therapies have demonstrated important value in improving patient survival [189]. Therefore, it is crucial to select appropriate indicators to predict treatment sensitivity, as well as to monitor variations in patients with RCC before and after treatment in real time to evaluate their efficacy. In the last 5 years, several clinical trials of liquid biopsy have focused on the monitoring of surgery, ICI, and anti-angiogenic therapy (Table 3). Song et al. showed that the number of CTCs decreased sharply after surgery in patients with RCC, with a more significant reduction in the pre-operative high CTC group (21 of 24 patients, 87.5%) than in the low CTC group (9 of 17 patients) [26]. Haga et al. showed that the number of postoperative CTCs was significantly correlated with tumor diameter (*P* = 0.0004) and surgical approach (*P* = 0.016), and pre-operative CTCs were significantly higher in patients with stage IV disease [190]. By classifying CTCs into epithelial CTCs, mesenchymal CTCs, and mixed CTCs and analyzing their Beclin-1 expression in patients with mRCC, Wang et al. reported that Beclin-positive mixed CTCs at 6 and 12 months postoperatively were significantly higher than pre-operative and 6 months postoperative CTCs, respectively [25]. This implies that CTCs could be a useful tool for monitoring postoperative metastasis and recurrence. In addition, cell-free nucleic acids have obvious potential for monitoring postoperative outcomes. Plasma GAPDH and hTERT cfDNA levels decreased significantly postoperatively and correlated with the risk of postoperative progression and death [191]. One study reported that each fg/mL of postoperative GAPDH cfDNA and hTERT cfDNA increased the risk of progression by 1.04 and 1.2 in Patients with RCC, respectively. In addition, each fg/mL of postoperative GAPDH cfDNA increased the risk of progression by 14.9 in Patients with RCC with metastasis. However, this study showed that postoperative GAPDH cfDNA and hTERT cfDNA levels were not independent risk factors in Patients with RCC without metastasis, and their levels at 1 year post-operation were not correlated with patient OS or PFS. Moreover, cell-free miR-15a levels decreased remarkably in patients undergoing nephrectomy. On the eighth postoperative day, the mean level decreased by 99.53% (*P* < 0.01) [169]; interestingly, the expression level correlated with tumor size. Combined with its diagnostic value, miR-15a may be a key molecule in liquid biopsies of RCC. As a traditional marker in liquid biopsies, plasma protein KIM 1 can also predict postoperative risk. Xu et al. showed that high postoperative baseline levels of KIM 1 were significantly associated with shorter disease-free survival (DFS) and OS in patients [192]. Adding it as a complementary measure to the SSIGN score or the UISS score improved the monitoring ability of postoperative metastasis (likelihood ratio test *P* = 0.078 and *P* = 0.0022, respectively). In addition, the levels of exosomal miR-210 and miR-1233 significantly decreased postoperatively (*P* = 0.004 and *P* = 0.008, respectively) [153].

**Table 3:** Monitoring treatment and predicting response by liquid biopsy in recent 5 years

	Region	Year	Detected Abnormality	Sample	Detection Method	Cohorts	Practice in clinical	Result	Ref
CTC	China	2021	CTC counts	Peripheral blood	CTC-BIOPSY system	41 RCC patients	Monitoring the postoperative condition of patients	• In the high CTC group, CTC counts decreased in 21 of 24 (87.5%) patients 1 week after surgery compared with the low CTC group (52.9%)	[26]
	USA	2021	PD-L1	Peripheral blood	VERSA Platform, Immunofluorescence	20 RCC patients treated with ICI	Evaluating the responses of ICI therapy	• PD-L1 expression in CAXII single positive CTC correlates with the efficacy of ICI treatment. Detection of progressing patients with ICI therapy: AUC 0.77, sensitivity 67% and specificity 88%	[24]
			HLA-1	Peripheral blood	VERSA Platform, Immunofluorescence	22 RCC patients treated with TKI	Evaluating the responses of TKI therapy	• PD-L1 expression in CAXII single positive CTC correlates with the efficacy of TKI treatment. Detection of progressing patients with TKI therapy: AUC 0.83, sensitivity 100% and specificity 88%	
	Japan	2020	CTC counts	Peripheral blood	FISHMAN-R flow cytometer	54 RCC patients treated with NE or RE	Evaluating the responses of surgery	• Postoperative CTCs was significantly correlated with tumor diameter (*P* = 0.0004) and surgical approach (*P* = 0.016)	[189]
	China	2019	Beclin-1	Peripheral blood	Can Patrol CTC enrichment technique, RNA ISH	58 metastasis-free RCC patients 11 metastasis RCC patients	Monitoring the postoperative metastasis	• Beclin1-positive epithelial CTCs in the metastatic group at 12 months postoperatively was significantly higher than preoperatively • Beclin1-positive mesenchymal CTC in the metastatic group at 6 months postoperatively was significantly higher than preoperatively • Beclin1-positive mesenchymal CTC in the metastatic group at 6 months postoperatively was significantly higher than preoperatively; at 12 months postoperatively was significantly higher than 6 months postoperatively and preoperatively	[25]
ctDNA/ cfDNA	Italy	2022	TP53	Plasma	NGS	12 mccRCC patients with immunotherapy 36 mccRCC patients with TKI therapy	Evaluating the responses of TKI and immune therapy	• The most frequently mutated genes in cfDNA were TP53 (43%) and PDGFRA (21%), followed by mTOR, PI3K, BRAF etc. The used NGS panel did not include VHL • Patients with at ctDNA > 0.883 ng/μl had a shorter PFS and OS versus those with ctDNA > 0.883 ng/μl in overall population (*P* < 0.001, *P* < 0.008, respectively). The results were consistent with patients treated immunotherapy and TKI separately (*P* < 0.0365, *P* < 0.0035, respectively) • Patients with TP53 mutation have a shorter PFS than those who do not (*P* = 0.04) • Comprehensively evaluated of both ctDNA level and TP53 mutation status, patients with high cfDNA and mutated TP53 had the worst PFS, while patients with low cfDNA and no TP53 mutations had the longer PFS (*P* = 0.004)	[51]
							Predicting the best response to TKI and immunotherapy	• cfDNA level was associated with best response in the overall population (*P* = 0.006), which is consistent with patients with immunotherapy (*P* = 0.004) and TKIs (*P* = 0.003) • cfDNA cut point of ≥ 2.19 ng/μl for early progressors: Youden’s 0.75, sensitivity 100%, specificity 75% • cfDNA cut point of ≤ 1.35 ng/μl for long progressors: Youden’s 0.556, sensitivity 78%, specificity 78%	
	Japan	2022	VHL, TP53, ATM, MET	Plasma	NGS	11 ccRCC patients with ICI therapy	Predicting response to ICI therapy in mRCC patients	• The commonly mutated genes were VHL (30.0%), TP53 (20.0%), ATM (10.0%), and MET (10.0%) • The coincidence rate of VHL (9 of 14 patients), TP53(2 of 14 patients) and MET (2 of 14 patients) between plasma ctDNA and tumor tissue DNA is 55.6%, 100%, 50%, respectively • For ICI-treated patients, ctDNA decreased in 4 of 5 responders and increased in 5 of 6 non-responders. A longer PFS is showed in the ctDNA-decreased group than ctDNA-increased group	[47]
	Spain	2021	GAPDH, hTERT	Plasma	qPCR	82 RCC patients 20 healthy controls	Evaluating surgery effects	• After nephrectomy, the mean level of GAPDH cfDNA was 16.9 fg/ml, which was significantly lower than preoperative level (29.3 fg/ml, *P* < 0.0001)	[190]
							Predicting patients' risk of death	• Univariate Cox Regression analysis showed that each fg/ml of GAPDH cfDNA increased the risk of progression by 14.8 postoperatively in mRCC patients • Each fg/ml of GAPDH cfDNA and hTERT cfDNA increased the risk of progression by 1.04 and 1.23 postoperatively, respectively	
	Japan	2019	VHL, TP53, mTOR, TSC1, BAP1et al	Plasma	NGS ddPCR	53 RCC patients	Monitoring responses to surgery and TKI therapy in RCC patients	• The mutant allele frequency (MAF) of VHL, TP53 and other ctDNA decreased postoperatively, which reflected the changes of tumor burden • Patients with short fragment sizes of cfDNA showed significantly worse responsiveness (*P* = 0.011). For TKI-treated patients, positive ctDNA was significantly associated with weaker effect (*P* = 0.049), and short fragment sizes of cfDNA tended to be associated with worse outcome (*P* = 0.090)	[50]
cfRNA	Ukraine	2018	miR-15a	Urine	qPCR	52 RCC patients 15 oncocytoma patients 15 healthy controls	Evaluating surgery effects	• The tumor size related to the expression of miR-15a (Pearson correlation coefficient 0.873) • The mean expression of miR-15a in patients with nephrectomy decreased by 99.53% (*P* < 0.01) on the 8th day postoperatively	[169]
	China	2018	lnc-GIHCG	Serum	qPCR	20 RCC patients with total nephrectomy	Evaluating surgery effects	• Serum GIHCG level significantly decreased in postoperatively compared than preoperatively (*P* < 0.001)	[173]
Protein	USA	2021	KIM-1	Plasma	microbead-based assay	418 ccRCC patients with total nephrectomy	Predicting patients' postoperative survivals	• Higher post-nephrectomy baseline of KIM-1 was related to worse DFS, with a survival time ratio of 0.65 (in univariable lognormal AFT model) and 0.56 (in variable lognormal AFT model) for the 75th vs 25th percentile of baseline KIM-1 (*p* = 0.0004, *P* < 0.001, respectively) • Higher post-nephrectomy baseline was related to worse OS in a multivariable AFT model, with a survival time ratio 0.71 for 75th vs 25th percentile of KIM-1 (*P* < 0.001)	[191]
							Predicting patients' postoperative recurrence risk	• Added to either the SSIGN score or the UISS score, baseline KIM 1 improved the predictive value for recurrence after nephrectomy of both models (likelihood ratio test *p* = 0.078, *p* = 0.0022, respectively)	
	UK	2021	CA9, HGF, MET, Gas6, Ax1, VEGF, VEGFR2, IL-8	Plasma	Luminex assay platforms Elisa	330 advanced RCC patients with cabozantinib 330 advanced RCC patients with everolimus	Predicting the survival and responses of cabozantinib	• The multivariable analysis showed that baseline levels of HGF were independent prognostic biomarker of PFS for cabozantinib, and HGF, GAS6, VEGF were independent prognostic biomarker for OS with cabozantinib • Decrease of AXL level were independent prognostic biomarker of PFS for cabozantinib, and decrease of levels of HGF, GAS6 were both independent prognostic biomarker for improved OS with cabozantinib	[193]
							Predicting the survival and responses of everolimus	• The multivariable analysis showed baseline levels of HGF were independent prognostic biomarker of longer PFS for everolimus. No biomarkers were independently prognostic for OS with everolimus • Decrease of HGF level were independent prognostic biomarker of PFS and OS for everolimus	
	USA	2021	23 angiokines, including Ang-2, CD-73, HER-3, HGF, IL-6, OPN etc	Plasma	SP-X imaging and analysis system from Quanterix Ella System (Protein Simple)	53 non-ccRCC patients with everolimus 46 non-ccRCC patients with sunitinib	Predicting the survival and treatment benefit of RCC therapy	Exception of HER-3, SDF-1, TGFb-R3 and BMP-9, higher angiokine levels were associated with worse PFS (HR > 1) The univariate analysis showed that exception of HER-3, SDF-1, TGFb-R3, VEGF-R1, and VEGF-R2, higher angiokine levels were associated with worse OS (HR > 1) The multivariable analysis showed that HGF, OPN, TIMP-1, TSP-2, and VCAM-1 were independently associated with OS with HRs of 1.5, 1.3, 1.7, 1.8, and 2.2, respectively None of the angiokines are statistically significant to predictive benefit for patients receiving either sunitinib or everolimus	[122]
	Spain	2021	CXCL10, CXCL11, HGF, IL-6	Serum	The Luminex’s xMAP Technology multiplex system	51 mRCC patients with sunitinib therapy 4 mRCC patients with pazopanib therapy 5 mRCC patients with both	Predicting patients' survivals with anti-angiogenic therapy	• High basal HGF levels (over 649.1 pg/mL) were significantly correlated to worse PFS (*P* = 0.003) and OS (*P* = 0.0034). A reduction of HGF levels during the treatment was related to a lower PFS (*p* = 0.017), but not with OS • CXCL11 levels were significantly higher in patients who did not respond to treatment (*P* < 0.05) than in those who responded. High levels of CXCL11 (above 39.4 pg/mL) were significantly related to shorter PFS (*P* = 0.0003) and OS (*P* = 0.001). Patients with a reduction of CXCL11 levels after 3 months treatment had a significantly lower OS (*P* = 0.027), but not PFS	[192]
	France	2021	SAA2, CFB	Plasma	Elisa	59 mRCC patients with sunitinib or bevacizumab treatment	Predicting survivals of mRCC patients with TKI therapy	• The levels of SAA2 and CFB can subdivide the cohort with anti-angiogenic treatment into 3 different groups according to PFS and OS: CFB low/SAA2 low (PFS 13.23 months, OS: 20.8 months), CFB low/SAA2 high or CFB high/ SAA2 low (PFS 9.87 months, OS 16.52 months), and CFB high/SAA2 high (PFS 2.8 months, OS 8.33 months)	[186]
	USA	2021	30 cytokines, including IL-6, IL-1RA, CSF, IFN-γ, IL-12, VEGF etc	Plasma	Luminex FLEXMAP 3D System	33 mccRCC patients with immunotherapy 23 mccRCC patients with TKI therapy	Predicting and monitoring response to ICI therapy in mRCC patients	• 17 patients are identified in clinical benefits (CB) group and 16 patients are identified in non-clinical benefits (NCB) group • No significant cytokine differences were observed between CB and NCB patients	[195]
							Predicting and monitoring response to TKI therapy in mRCC patients	• 13 patients are identified in clinical benefits CB group and 10 patients are identified in non-clinical benefits NCB group • Patients in CB group had lower median levels of IL-6 (8.4 vs 13.5 pg/mL, *p* = 0.02), IL-1RA (178 vs 248 pg/mL, *p* = 0.03), and G-CSF (23.9 vs 38.3 pg/mL, *p* = 0.02) compared with patients in NCB group in pretreatment	
	USA	2020	Ang-1, Ang-2, HGF, CXCL10, IL2, IL6, IL8, IL10, CXCL9, NGAL, OPN, TGFb, VEGF etc	Serum	Luminex instrument	52 advanced RCC patients with combined axitinib and pembrolizumab treatment	Predicting the survival and treatment benefit of RCC therapy	• Higher baseline of CXCL10 was correlated with objective response rate (ORR) (unadjusted P value = 0.0197) • Lower EOT level of CEACAM1, GRO-a, HGF, and TIMP-1 was correlated with objective response rate (ORR) (unadjusted P value = 0.0026, 0.0495, 0.0112, 0.0044, respectively) • At baseline, CEACAM1 levels ≥ median were associated with better PFS (*P* = 0.085). C2D1, GRO-a and HGF levels were associated with better PFS (*P* = 0.0034, *P* < 0.001, respectively). At EOT, HGF and TIMP-1 levels < median were associated with better PFS (*P* = 0.0034, 0.014, respectively)	[194]
	Italy	2020	PD-1, PD-L1, BTN3A1	Plasma	Elisa	Testing cohort: 21 mccRCC patients with nivolumab treatment Validating cohort: 20 mccRCC patients with nivolumab treatment 15 localized ccRCC patients	Predicting survivals of mccRCC patients with immunotherapy	• The mean pre-treatment levels of plasma ICs in long-responder group (> 18 months) was significantly higher than all patients (PD-1: 13.25 vs. 2.00 ng/mL, *p* = 0.01; PD-L1: 1.09 vs. 0.64 ng/mL, *p* = 0.02; BTN3A1: 11.03 vs. 6.84 ng/mL, *p* = 0.03) • High level of plasma PD-1 (> 2.11 ng/mL), PD-L1 (> 0.66 ng/mL) and BTN3A1 (> 6.84 ng/mL) were correlated to a shorter median PFS (PD-1: 20.7 vs. 6.9 months, *P* < 0.0001; PD-L1: 19 vs. 9 months, *P* < 0.0001; BTN3A1: 17.5 vs. 8.4 months, *p* = 0.002) • After 18 months of immunotherapy, plasma PD-1 and PD-L1 levels were lower than baseline in patients with FPS longer than 18 months (PD1: 1.23 vs. 13.25 ng/mL; PD-L1: 0.73 vs. 1.09 ng/mL) • The predictive value of PD1, PD-L1 and BTN3A1 in validating cohort: PD1: AUC = 1.0, *P* < 0.001; PD-L1: AUC = 0.944, *P* < 0.001; BTN3A1: AUC = 0.833, *P* < 0.03	[187]
Exosome	China	2018	Exsomal miRNA miR-210, miR-1233	Serum	Total exosome isolation reagent, EpCAM isolation beads, Flow cytometry	10 ccRCC patients received surgical tumor removal	Monitoring responses to surgery	• The levels of exosomal miR-210 and miR-1233 were significantly lower in postoperative than in preoperative samples (*p* = 0.004, *p* = 0.008, respectively)	[153]

In addition to surgery, the use of liquid biopsy in predicting the responses to tyrosine kinase inhibitors (TKIs) and ICI therapy deserves attention. Bade et al. highlighted the use of CTCs in the evaluation of responses to ICI and TKI therapy [24]. This study divided patients receiving TKI and ICI therapy into responding and progressive groups and differentiated them using characterized CTCs. The results showed that HLA-1 and PD-1 expression in CAXII single-positive CTCs could effectively identify patients in the progressive phase (AUC = 0.77 and 0.83, respectively), thereby predicting sensitivity to adjuvant therapy and helping to select a suitable drug. A study in 2022 identified the most commonly mutated genes in 11 patients with ccRCC receiving ICI therapy, including VHL (30.0%), TP53 (20.0%), ATM (10.0%), and MET (10.0%). They then explored the concordance between plasma ctDNA and tumor DNA [47]. The results showed that ctDNA decreased in four of five responders and increased in five of six non-responders. Interestingly, patients with elevated ctDNA levels had shorter PFS than those with decreased ctDNA levels. Another recent study also evaluated the mutation status of patients, showing that TP53 had the highest mutation frequency (43%), which was significantly higher than the former results. This study showed that ctDNA levels could not only predict survival time after treatment but also distinguish early response from long-term response; they could also be combined with TP53 mutation status to assess patient risk [51]. Yamamoto et al. concluded that the frequency of ctDNA mutations was significantly reduced postoperatively. In addition, shorter size and higher levels of ctDNA were associated with adverse effects and sensitivity in patients receiving TKI therapy (*P* = 0.049 and *P* = 0.090, respectively) [50].

Protein analysis is widely used to assess and monitor the efficacy of ICI and anti-angiogenic therapy. To assess the response to anti-angiogenic therapy, Esteban et al. explored protein levels in patients with mRCC receiving anti-angiogenic therapy, including sunitinib and pazopanib [193]. High levels of HGF were significantly associated with poorer PFS (*P* = 0.003) and OS (*P* = 0.0034) in patients receiving anti-angiogenic therapy. HGF levels were higher in treatment-effective patients than in treatment-ineffective patients at baseline (*P* < 0.05), but there was no difference after three months of treatment. Similarly, CXCL11 levels were significantly associated with poor PFS (*P* = 0.003) and OS (*P* = 0.0034) and non-response to anti-angiogenic therapy (*P* < 0.05). The reduction in CXCL11 after treatment was associated with shorter OS in patients (*P* = 0.027) but not PFS. In addition, Cooley et al. classified patients receiving anti-angiogenic therapy (sunitinib and bevacizumab) into three categories according to SAA2 and CFB levels [187]. The results showed that the CFB low/SAA2 low group had the best survival (PFS: 13.23 months, OS: 20.8 months); in contrast, the CFB high/SAA2 high group had the worst survival (PFS: 2.8 months, OS: 8.33 months). Moreover, as the direct target of ICI therapy, the level of immune checkpoints in plasma is closely related to the response to ICI treatment [188]. Patients who responded for a long time (> 18 months) to ICI therapy had significantly higher plasma levels of immune checkpoints; however, patients with RCC with high levels of immune checkpoints had a lower median survival. This result indicates a strong association between plasma immune checkpoint proteins and ICI therapy. As a fundamental component of circulating proteomics, a large number of angiokines and chemokines are involved in the evaluation of cancer therapy. Several results have shown that HGF can significantly differentiate the survival time and status of patients with RCC receiving anti-angiogenic therapy and immunotherapy [117, 122, 193–195]. Currently, HGF is demonstrating its clinical potential as a biomarker in a variety of drugs, including cabozantinib [194], sunitinib [122, 193], axitinib [195], pazopanib [193], pembrolizumab [195] and everolimus [122, 194]. In addition, OPN and IL-6 have been widely reported and may be considered biomarkers for therapeutic selection and evaluation[122, 196]. Many other cytokines have been reported with the difference of baseline levels and dynamic changes during treatment between patients with good versus poor prognosis, but there is a lack of consistency between the results. It is worth noting that there are few studies focusing on the combined use of drugs at present, and all of them concentrated on the combined use of antiangiogenic therapy and immunotherapy, while there is a lack of research on the combined use of multiple anti-angiogenic therapy or immunotherapy. Besides, Daniela Vargová et al. analyzed urinary cytokines in 60 RCC patients [197]. The results showed that the baseline level of platelet-derived growth factor (PDGF), IL-15, MIP-1ß, etc. were higher in patients with RCC than healthy controls, and the cytokines decreased at the third day postoperatively. However, the study did not do a more in-depth analysis.

### Limitations and prospects

Commonly used metrics in RCC include CTCs, ctDNA/cfDNA, cfRNA, exosomes, and tumor-derived metabolites or proteins. However, these indicators have several limitations. The low level of CTCs in the blood makes their capture and analysis very difficult. Moreover, they have a relatively low diagnostic value. With the popularity of NGS, ddPCR, and methylation analysis, ctDNA/cfDNA is becoming less expensive and more accurate and is widely used in various clinical practices. However, it is still difficult to distinguish ctRNAs from cfRNAs based on genomic alterations. Among cfRNAs, miRNAs are the main molecules used for liquid biopsies. However, the complexity of the miRNA regulatory network makes it difficult to explain the mechanism underlying its role as a biomarker. In addition, many studies have pointed out that the standardization of miRNA analysis, including analytical conditions and methods, still needs to be determined [198, 199]. Although considered ideal components for liquid biopsy, cohort studies of lncRNAs, circRNAs, and piRNAs are insufficient to fully demonstrate their clinical value. Similarly, despite the recent increase in studies on urinary metabolites in liquid biopsies, few studies have been able to reveal the metabolic pathway changes involved, and the metabolites screened showed low consistency. Protein is a traditional molecule used for liquid biopsy, and many automated analytical systems or platforms have emerged in recent years, attempting to detect target proteins with high sensitivity and specificity. However, their accuracy needs to be tested in larger cohorts. Among them, multiple cytokines were used to assess and monitor treatment, and HGF, OPN and IL-6 had shown their predictive abilities. However, there were still a large number of cytokines whose predictive outcomes were inconsistent, as demonstrated in the evaluation of responses of everolimus. In addition, fewer studies have been conducted on cytokines in urine, and current studies lack sufficient attention to the correlation between cytokines in body fluids and the molecular phenotype of tumor cells. Furthermore, the role of exosomes in cancer has received increasing attention, but a standard isolation method that can guarantee both the purity and availability of the product is still lacking.

At the same time, we should recognize that almost all current studies on therapeutic response assessment have focused on a single anticancer drug, resulting in a failure to explore the clinical value of liquid biopsy in treatment regimens based on the combination of multiple drugs. The combined application of immunotherapy and targeted therapy has become clinically prevalent, and further studies should focus on this aspect. In addition, few studies have discussed the possibility of using liquid biopsy with other conventional clinical tests such as imaging tests. As a non-invasive test with potential for dissemination, a novel risk score or guideline, including liquid biopsy with other clinical tests, should be established. Meanwhile, the frontier areas of artificial intelligence and machine learning should be noted and used in combination with liquid biopsies to improve reliability and performance. For the purpose, a global integrated information center should be established to provide comprehensive information on liquid biopsies. Besides, we believe that commercially available testing platforms or kits should be further developed as the ultimate liquid biopsy products to accelerate promotion it to clinical practice. Finally, owing to the clonal diversity of tumors, the genome, transcriptome, proteome, and metabolome have significant interactions between primary and metastatic tumors. This makes it difficult to precisely guide treatment by analyzing a single molecule. Single-cell sequencing can reduce the impact of heterogeneity by identifying the molecular phenotype of captured tumor cells in peripheral blood, and is currently being used in several studies [118, 200]. This also indicates that the combined analysis of multiple liquid biopsy components can yield more information to further facilitate precision medicine.

However, despite several limitations, the advantages of liquid biopsy are obvious and cannot be ignored. A growing number of clinical trials are underway in RCC to improve the accuracy and reliability of liquid biopsy and address these issues. Here, we present the current ongoing clinical trials of liquid biopsy in RCC (Table 4). We believe that future research on liquid biopsy will focus on the following issues: (1) establishing better medical information systems that collect more clinical data to support high-quality trials with large patient cohorts; (2) developing new technologies and analytical platforms to improve measurement efficiency and reduce costs; (3) finding biomarkers with higher sensitivity and specificity; (4) standardizing biomarker isolation techniques and establishing guidelines to increase objectivity and reproducibility of studies; and (5) using a combination of liquid biopsy and other clinical tests and establishing new risk scores or guidelines.

**Table 4:** Currently ongoing clinical trials focused on liquid biomarkers in renal cell carcinoma

NCT Number	Liquid biopsy component	Study Name	Type	Country and number of patients	Recruiting Status	Brief summary
NCT04883827	CfDNA	Monitoring Disease Burden and Biology Using Tumor Cell Free DNA in Metastatic Kidney Cancer	Observational	United States;150	Recruiting	This study will assess whether DNA released by kidney cancer into the blood stream and urine of patients can be used to monitor tumor burden and tumor response to treatment in patients receiving immunotherapy
NCT04197414	ctDNA	Development of Urologic Registry for Personalized Medicine in Patients with Urological Malignancy by Analyzing Circulating Tumor DNA	Observational	Korea; 3000	Recruiting	This study aims to explore the usefulness of ctDNA in plasma and urine for the detection of urologic malignancies, the monitoring of disease progression and the assessment of treatment response
NCT05059444	ctDNA	ORACLE: Observation of Residual Cancer with Liquid Biopsy Evaluation	Observational	United States; 1000	Recruiting	This study aims to demonstrate the ability of a new ctDNA assay developed by Guardant Health to early detect recurrence in patients with solid tumors (including RCC)
NCT03702309	cfDNA; cfRNA	Liquid Biopsy Evaluation and Repository Development at Princess Margaret	Observational	Canada; 2500	Recruiting	This study aims to use circulating cell-free nucleic acids, including cfDNA and cfRNA, as a means to non-invasively assess multiple tumor progression and treatment response at multiple time points in a patient's disease course
NCT04891055	CTC; miRNAs	Prospective Translational Study Investigating Predictors of Outcome in Metastatic Renal Cell Carcinoma Patients Treated with Nivolumab (I-Rene Trial)	Observational	Italy; 90	Recruiting	The study aims to evaluate whether circulating miRNAs or CTCs may be a potential predictor of clinical response and disease progression in metastatic RCC patients treated with Nivolumab
NCT03667885	DNA and mRNAs	Non-Invasive Diagnostics of Small Renal Masses	Observational	Denmark; 160	Recruiting	Studies 1 and 2 were designed to look for circulating biomarkers in the form of DNA and mRNA contained in microvesicles secreted into the blood by RCCs and to find changes in biomarker levels after surgery
NCT04946266	LncRNA-MFI2-AS1	Prospective Validation of the Prognostic Value of Long Non-coding MFI2-AS1 RNA in Localized Clear Cell Kidney Cancers	Observational	France; 260	Recruiting	This study aims to detect the expression of LncRNA-MFI2-AS1 in the plasma of Localized Clear Cell Kidney Cancers to explore its use as a biomarker for pre-tissue analysis diagnosis and patient follow-up
NCT04053855	Urinary exosomes	Evaluation of Urinary Exosomes Presence from Clear Cell Renal Cell Carcinoma	Observational	France; 100	Recruiting	This study aims to develop a reliable technique to detect tumor exosomes in the urine of ccRCC patients and to provide a new liquid biopsy tool for their early diagnosis
NCT05060783	Glycosaminoglycans	Renal Cancer Detection with Liquid Biopsy	Observational	Denmark; 200	Recruiting	This study aims to explore the diagnostic role of alterations in plasma and urine glycosaminoglycans for RCC
NCT03628859	Intracellular cytokines	BIOREN (Predictive BIOmarkers in Metastatic RENal Cancer)—A Translational Study on Immunotherapy for Metastatic Renal Cancer	Observational	France; 30	Recruiting	This study aims to characterize the genetic background of RCCs and their immune environment, to try and identify biomarkers of response and to better understand the mechanisms of resistance to nivolumab in RCC
NCT03185039	MMP2 and MMP9	Predictive Impact of MMP2 and MMP9 Levels for Patients with Metastatic Kidney Cancer Treated with Anti-angiogenic Agents	Interventional	France; 50	Recruiting	This study aims to explore plasma levels of MMP2 and MMP9 as predictive biomarkers for treatment with 2 anti-angiogenic drugs (sunitinib or pazopanib) in patients with metastatic kidney cancer
NCT05214885	CA9、NDUFA4L2、ANGPTL4、HILPDA and EGLN3	Novel Biomarkers of Hypoxia and Metabolism in Clear Cell Renal Cell Carcinoma	Observational	China; 300	Recruiting	This study aims to detect mRNA and protein levels of five hypoxia- and metabolism-related molecules in blood or urine samples to explore specific tumor biomarker profiles for clinical diagnosis, assessment of ccRCC recurrence, metastasis and prognosis
NCT04113486	NMAP	Expression Levels of Nicotinamide Metabolism-related Protein (NMAP) in Newly Diagnosed Renal Cancer and Non-renal Cancer Populations	Observational	China; 400	Recruiting	This study aims to observe the difference between NMAP serum levels in primary diagnosed RCC patients and controls and to plot the ROC curve and establish appropriate cut-off values
NCT05285579	Immune-related circulating biomarkers	Predictive Role of Circulating Biomarkers Involved in Angiogenesis in Metastatic Kidney Cancer in the Era of New Therapeutic Associations: Immunotherapies, Anti-angiogenic	Observational	France; 100	Not yet recruiting	This is a multicenter, exploratory, prospective study to identify angiogenesis and immune-related biomarkers predictive of progression free survival in patients with metastatic or advanced RCC treated by a combination of immunotherapy and antiangiogenic
NCT04006405	Glycosaminoglycans	AURORAX-0087A: Glycosaminoglycan Scores for Surveillance of Recurrence in Leibovich Points ≥ 5 Non-metastatic Clear Cell Renal Cell Carcinoma	Observational	United States; 280	Recruiting	This is an observational prospective, multicenter, diagnostic test cohort study which aims to predict disease recurrence in non-metastatic ccRCC using glycosaminoglycans scores in blood and urine
NCT04712305	Metabolomics and Proteomics	Urine Metabolomics and Proteomics Profiling to Predict the Responses and Adverse Events of Immuno-Oncology-based Therapy in Patients With Advanced Renal Cell Carcinoma	Observational	Taiwan; 400	Recruiting	The study aims to identify urinary metabolite and protein markers that can predict anti-tumor efficacy and adverse events in subjects receiving Immuno-Oncology-based therapies for metastatic RCC
NCT05112627	Serum immune markers	Immunophenotyping in Metastatic Renal Cell Carcinoma Patients Receiving Ablative Therapy	Observational	United States; 45	Recruiting	This study aims to evaluate serum immune markers and peripheral blood mononuclear cell characteristics in patients with metastatic RCC treated with SBRT or PCA, and the impact on their overall distant disease progression

## Conclusion

Currently, liquid biopsy has shown remarkable potential and value for many cancers. In RCC, owing to its advantage of being non-invasive, liquid biopsy can be used for screening in healthy populations or for differential diagnosis of masses of unknown pathological types. Furthermore, for patients with confirmed disease, liquid biopsy can predict survival and progression risk, thus identifying high-risk patients for future research. In addition, liquid biopsy can predict the efficacy of different adjuvant therapies for patients so that personalized treatment plans can be made to practice precision medicine. Additionally, following surgical or adjuvant treatment, liquid biopsy can be used to evaluate the effectiveness of treatment and predict the risk of recurrence or metastasis by continuously monitoring relevant indicators. Owing to its wide range of applications and substantial clinical value, an increasing number of clinical trials are being conducted to find suitable biomarkers and develop more efficient and affordable extraction techniques. Compared to traditional techniques, technological innovations and the maturation of commercial platforms in recent years have improved the isolation efficiency and purity of biomarkers, and many biomarkers have shown their potential. Therefore, despite many unresolved limitations, we believe that liquid biopsy has substantial clinical value and represents a future development direction for cancer diagnosis, prognostic assessment, and treatment monitoring.

## Data Availability

Not applicable.

## Abbreviations

RCC: Renal cell carcinoma
CTC: Circulating tumor cells
cfDNA: Cell-free DNA
ctDNA: Cell-free tumor DNA
cfRNA: Cell-free RNA
ccRCC: Clear cell renal cell carcinoma
mTOR: Mammalian target of rapamycin
RTK: Receptor tyrosine kinase
ICI: Immune checkpoint inhibitor
EpCAM: Epithelial cell adhesion molecule
CAIX, CAXII: Carbonic anhydrase IX, XII
CK: Cytokeratin
CD: Clusters of differentiation
FISH: Fluorescence in situ hybridization
VHL: Von Hippel-Lindau
GAs: Genomic alterations
HIF: Hypoxia inducible factor
RT-qPCR: Real-Time quantitative Polymerase Chain Reaction
dPCR: Digital PCR
ddPCR: Droplet digital PCR
NGS: Next-Generation Sequencing
cfMeDIP-seq: Cell-free methylated DNA immunoprecipitation and high-throughput sequencing assay
EVs: Extracellular vesicles
miRNAs: MicroRNAs
FH: Ferredoxin hydratase
SDH: Succinate dehydrogenase
MS: Mass spectrometer
LC–MS: Liquid chromatography-MS
GC–MS: Gas chromatography-MS
MALDI-MS: Matrix assisted laser desorption/ionization-MS
UPLC-MS: Ultra-performance LC–MS
HS–SPME–GC–MS: Headspace solid-phase microextraction coupled with GC–MS
NMR: Nuclear magnetic resonance
2HG: 2-Hydroxyglutarate
ELISA: Enzyme linked immunosorbent assay
CEA: Carcinoembryonic antigens
GRP78: 78 KDa glucose-regulated protein
PD-1: Programmed cell death protein 1
PD-L1: Programmed cell death 1 ligand 1
KIM-1: Kidney injury molecule 1
NGAL: Neutropil gelatinase-associated lipocalin
TME: Tumor microenvironment
ILVs: Intraluminal vesicles
MVBs: Multivesicular bodies
EE: Early endosomes
BAP1: BRCA1-associated protein 1
PBRM1: Recombinant polybromo 1
DMRs: Differentially methylated regions
ROC: Receiver operating characteristic curve
AUC: Area under ROC
MET: Epithelial-mesenchymal transition
qMSP: Methylation-specific quantitative PCR
ZEB2: Zinc finger E-box binding homeobox
BLCA: Bladder cancer
hPG80: Human Circulating Progastrin 80
PLIN-2: Perilipin-2
OS: Overall survival
PFS: Progression-free survival
PCF: Proportion of cfDNA fragments
HR: Hazard ratio
SAA2: Serum Amyloid A2
CFB: Complement Factor B
TKI: Tyrosine kinase inhibitor
SRMs: Small renal masses
